# Physical Exercise-Induced Adult Neurogenesis: A Good Strategy to Prevent Cognitive Decline in Neurodegenerative Diseases?

**DOI:** 10.1155/2014/403120

**Published:** 2014-04-09

**Authors:** Suk-yu Yau, Joana Gil-Mohapel, Brian R. Christie, Kwok-fai So

**Affiliations:** ^1^Division of Medical Sciences, University of Victoria, 3800 Finnerty Road, Victoria, BC, Canada V8P 5C2; ^2^Department of Ophthalmology, The University of Hong Kong, 21 Sassoon Road, Pokfulam, Hong Kong; ^3^State Key Laboratory of Brain and Cognitive Science, The University of Hong Kong, 21 Sassoon Road, Pokfulam, Hong Kong; ^4^Department of Anatomy, Li Ka Shing Faculty of Medicine, The University of Hong Kong, 21 Sassoon Road, Pokfulam, Hong Kong; ^5^Guangdong-Hong Kong-Macau Institute of CNS Regeneration, Jinan University, 601 Huangpu Avenue West, Guangdong 5106032, China; ^6^Guangdong Key Laboratory of Brain Function and Diseases, Jinan University, 601 Huangpu Avenue West, Guangzhou 5106032, China

## Abstract

Cumulative evidence has indicated that there is an important role for adult hippocampal neurogenesis in cognitive function. With the increasing prevalence of cognitive decline associated with neurodegenerative diseases among the ageing population, physical exercise, a potent enhancer of adult hippocampal neurogenesis, has emerged as a potential preventative strategy/treatment to reduce cognitive decline. Here we review the functional role of adult hippocampal neurogenesis in learning and memory, and how this form of structural plasticity is altered in neurodegenerative diseases known to involve cognitive impairment. We further discuss how physical exercise may contribute to cognitive improvement in the ageing brain by preserving adult neurogenesis, and review the recent approaches for measuring changes in neurogenesis in the live human brain.

## 1. Introduction

Given the overwhelming evidence showing adult neurogenesis in the mammalian brain [1–5] and its potential role in cognitive function [6–11], the relationship between adult neurogenesis, cognitive deficits, and neurodegenerative diseases has become an emerging topic of interest. This is of particular relevance in the ageing population, given the increasing prevalence of cognitive deficits associated with neurodegenerative diseases. Therefore, manipulation of adult neurogenesis has currently been targeted as a potential treatment for ageing-related cognitive deficits.

**Figure 1:**
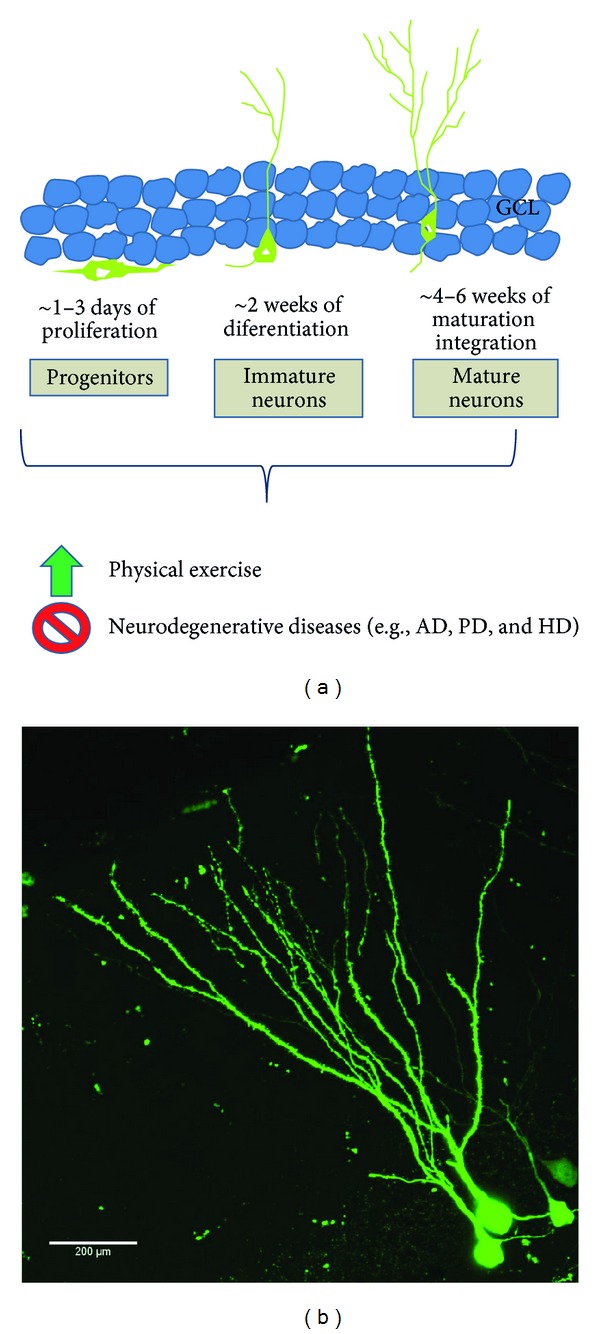
Development and integration of adult-born neurons in the dentate gyrus of the hippocampus. (a) The neural progenitors that are divided from neural stem cells start expressing either neuronal or glial phenotypes after just a few days of division. Newborn neurons gradually migrate from the subgranular zone (SGZ) into the granular cell layer (GCL) where they undergo maturation, followed by functional integration into the existing neural circuitry in the hippocampus. This process of hippocampal neurogenesis is known to be promoted by physical exercise and to be compromised in several neurodegenerative diseases such as AD, PD, and HD. (b) Confocal image of 4-week-old retroviral-labeled newborn neurons with green fluorescence protein (GFP) in the GCL (scale bar: 200 *μ*m).

Newborn neurons are mainly produced from neural stem cells in two neurogenic zones of the adult brain: the subventricular zone (SVZ)/olfactory bulb (OB) and the subgranular zone (SGZ) of the hippocampal dentate gyrus (DG) [12]. In the SVZ, neural stem cells give rise to committed progenitor cells that migrate through the rostral migratory stream (RMS) into the OB where they differentiate into local interneurons, specifically granular and periglomerular neurons. Adult neurogenesis in the hippocampus is more locally confined, when compared to neurogenesis in the SVZ. In the DG of the hippocampus, newborn neurons migrate just a short distance (approximately 20 to 25 *μ*m, two cell nuclei wide) from the SGZ to the granule cell layer (GCL), where they integrate into the existing circuitry [12]. A dividing progenitor cell gives rise to daughter cells which differentiate, migrate, and integrate integrate into the existing circuity. Their dendrites extend to the molecular layer of the DG while their axons project to the cornus ammonis (CA) 3 region, through the mossy fiber pathway [13] (Figure 1). Retroviral labeling of newborn cells with green fluorescent proteins has revealed that newborn neurons can form synaptic contact with its target cells by the third week of neuronal maturation [14]. Therefore, synaptic connections between the DG and the CA3 hippocampal subregions (which form the mossy fiber tract) can potentially be modified by changes in hippocampal neurogenesis. About 9000 new cells are generated each day in the rodent hippocampus (hundreds of thousands of cells each month, accounting for 6% of the total granule neuronal population) of which about 80–90% differentiate into neurons [15].

Clinical studies have confirmed that similar processes also occur in the corresponding regions of the human brain [16–19]. The first evidence of adult neurogenesis in the human brain came from a study showing the presence of positive staining for 5-bromo-2′-deoxyuridine (BrdU, a thymidine analog) in the SVZ and the DG region of postmortem brain sections from cancer patients who had received BrdU injections in life [5]. These findings have since then been confirmed and a recent study has revealed that approximately 700 new neurons are added to the adult human hippocampus each day [20]. However, adult neurogenesis is age dependent with the production of new neurons declining with age [21–28].

The hippocampus plays an integral role in the consolidation of declarative memory, as well as context dependent and spatial learning processes [29, 30] in both humans [31, 32] and rodents [33–36]. New hippocampal neurons are believed to contribute to the functioning of the hippocampus and there is evidence that they are recruited into hippocampal neuronal circuits known to be involved in spatial learning [9] and possess particular physiological properties that make them more susceptible to behavioral-dependent synaptic plasticity [11, 37, 38]. Thus, it is reasonable to speculate that these new neurons might be integral for hippocampal-dependent learning and memory [11], and in particular pattern separation [39–41]. In agreement with this hypothesis, numerous correlative studies have shown that hippocampal neurogenesis can be modulated by learning and behavioural experience [6, 42–45] and that a loss in hippocampal neurogenic function can adversely affect memory formation [7, 8, 10, 38, 46].

Using exercise training as an upregulator for hippocampal neurogenesis, an* in vivo* imaging study in humans has indicated a positive association between hippocampal-dependent cognitive performance and change of cerebral blood volume (CBV: served as an indirect measure of changes in hippocampal neurogenesis in the human brain) [47]. Furthermore, exercise intervention has been shown to improve performance in a neurogenesis-dependent cognitive test, the visual pattern separation task in human subjects [48]. In spite of the technical limitations associated with the direct measurement of neurogenesis in the human brain, these two studies have suggested that adult-born new neurons in the hippocampus might play a functional role in learning and memory in the human brain.

Neurodegenerative diseases such as Alzheimer's disease (AD), Parkinson's disease (PD), and Huntington's disease (HD) share the common characteristic of progressive loss of structure and/or function of neurons in the brain. Although neuronal degeneration predominantly affects specific neuronal populations (i.e., dopaminergic neurons in PD, striatal gamma-aminobutyric acid (GABA) ergic neurons in HD, and cortical and hippocampal neurons in AD), all these neurodegenerative diseases are characterized by a more or less severe loss of certain cognitive functions including learning and memory. Concomitantly, several lines of evidence have shown that adult hippocampal neurogenesis might be altered in these neurodegenerative processes [49, 50].

Physical activity has been repeatedly shown to improve cognition and prevent age-related cognitive decline in humans [51], particularly in individuals affected with certain neurodegenerative diseases [52, 53]. However, the underlying mechanisms responsible for the beneficial effects of physical exercise are still unclear. Nevertheless, animal studies have suggested that an increase in hippocampal neurogenesis may mediate, at least in part, the exercise-induced increase in cognitive function [54, 55].

Here, we review the functional role of adult neurogenesis in cognitive function and the emerging association between adult hippocampal neurogenesis and cognitive impairment in neurodegenerative diseases. We further discuss physical exercise-induced hippocampal neurogenesis and its relationship with cognitive improvement. Finally, we address the emerging techniques for measuring adult neurogenesis in live human brain.

## 2. Adult Neurogenesis in Learning and Memory

The functions of adult neurogenesis in the adult brain have been extensively investigated in the past decade. Numerous studies have suggested that neurogenesis in the DG may play an important role in hippocampal-dependent learning and memory [6–11, 56], as well as affective disorders such as depression and anxiety [56–59], while neurogenesis in the SVZ may be involved in olfactory learning and discrimination [60] and sexual behavior [61, 62].

Importantly, newly generated neurons have particular physiological properties that make them more susceptible to behavioral-dependent synaptic plasticity [11]. Using retroviral labeling of newborn neurons with green fluorescence protein, Toni and colleagues have demonstrated that newborn neurons could form synapses and receive synaptic input from existing neurons [14]. Furthermore, immature neurons exhibit a lower threshold for long-term potentiation (LTP) induction in response to theta-burst stimulation [37], which might be due to their specific membrane properties such as greater N-methyl-D-aspartate (NMDA) receptor sensitivity and calcium entry upon synaptic activation [63]. On the other hand, LTP has also been shown to induce adult hippocampal neurogenesis [64], which further strengthens the link between structural and functional hippocampal plasticity.

Since these newly generated neurons are linked to the functioning of the hippocampus, it is reasonable to speculate that they might play a role in mechanisms of hippocampal-dependent learning and memory. In agreement with this hypothesis, it has recently been shown that new neurons are indeed recruited into neuronal circuits involved in spatial learning and memory in the hippocampus [9]. Furthermore, other studies have shown that disrupting or ablating adult hippocampal neurogenesis results in impaired hippocampal-dependent learning and memory. Experimental reduction of adult neurogenesis impaired hippocampal-dependent trace eye-blink conditioning but not hippocampal-independent delay conditioning [7]. Similar results were obtained with other hippocampal-dependent tasks, including place-recognition tasks [46], contextual fear conditioning [8, 38], and a non-matching-to-sample task, which measured conditional rule learning and memory for specific events [8].

Although details of how newborn neurons modulate learning and memory are still unclear, recent findings have suggested that adult born neurons in the DG play a critical role in pattern separation, preventing memory interference from overlapping contexts [39, 65, 66]. Garthe and colleagues have demonstrated that inhibiting neurogenesis in mice results in impairments in the reverse protocol of the Morris Water Maze test (i.e., an increased preference for the old position of the hidden platform and failure to identify the new position). These results suggest that adult neurogenesis in the DG prevents memory interference from similar contexts, thus allowing formation of a new memory that is similar to a previously acquired one [67]. In agreement with this finding, two recent studies have demonstrated an association between lower levels of hippocampal neurogenesis and impairments in spatial pattern separation in mice [39, 68]. Conversely, exercised mice with enhanced neurogenesis perform better in spatial pattern separation tasks [66].

To test the hypothesis concerning the functional role of neurogenesis on pattern separation in the human brain, Dery and colleagues used the visual pattern separation task, a cognitive test that is believed to be neurogenesis-dependent and that uses some objects that are repeatedly presented across trials and some objects that are new but highly similar to previously presented ones. They observed a significant enhancement in performance on the visual pattern separation task together with lower depression scores in subjects who participated in exercise training a well-known enhancer of neurogenesis [48]. This finding corroborates the hypothesis that adult hippocampal neurogenesis may be involved in learning and memory in the human brain.

In summary, it is currently believed that hippocampal new neurons are required for the separation of events based on their spatial and temporal characteristics (a process that preserves the uniqueness of a memory representation), as well as space representation, long-term memory retention, and flexible inferential memory expression [69].

## 3. Altered Adult Neurogenesis in Neurodegenerative Diseases

The contribution of altered adult hippocampal neurogenesis to the cognitive deficits that are characteristic of various neurodegenerative conditions such as AD, PD, and HD is still not fully elucidated. Nevertheless, since alterations in adult neurogenesis have been repeatedly shown in various animal models of these disorders [70] (Table 1), it is speculated that cognitive decline in neurodegenerative diseases could be partly due to alterations in the neurogenic process. Within this scenario, therapeutic strategies such as physical exercise that can restore or increase adult neurogenesis might be of therapeutic value for the treatment of the cognitive deficits associated with these devastating neurodegenerative disorders.

### 3.1. Hippocampal Neurogenesis in Alzheimer's Disease (AD)

AD is manifested by progressive cognitive deterioration, memory loss, behavioural changes, and eventually dementia. At the pathological level, AD is characterized by acetylcholine depletion, the accumulation of amyloid (or senile) plaques, and the formation of neurofibrillary tangles (NFT), which can lead to neuronal loss by apoptosis particularly in the cortex and hippocampus and severe brain atrophy [71]. While the majority (95%) of cases of AD are sporadic, complex arrays of environmental and genetic factors have also been linked to the etiology of this disorder. Gene mutations in the presenilin (PS) 1 and/or 2 genes or the apolipoprotein (APO) E gene can increase the risk of developing AD [72, 73]. PS1 and PS2 are key components of *γ*-secretase, the enzyme responsible for cleaving the amyloid precursor protein (APP) into toxic amyloid-*β* (A*β*) peptides, the building blocks of senile plaques [74].

While the exact neurobiological mechanisms underlying the symptoms of AD are still unclear, severe neuronal loss in areas of the brain involved in learning and memory, such as the hippocampus and prefrontal cortex, is evident in the AD brain. Transgenic mouse models of AD show impairments in several hippocampal-dependent learning and memory tasks, such as spatial learning, object recognition, and contextual fear conditioning [75]. Additionally, adult hippocampal neurogenesis has been investigated in several of these models and contradictory results have been obtained [50, 76, 77]. Briefly, while a decrease in neurogenic function has been reported in transgenic or knock-in mice carrying the Swedish mutation in the APP gene [78–81], the PDAPP mutation [82], mutations in the PS1 gene [80, 81, 83, 84], as well as in double-transgenic mice for APP and PS1 [80, 81], and in triple-transgenic mice for APP, PS1, and tau protein [85], others have found increased hippocampal neurogenesis in transgenic mice that express APP with the Swedish and the Indiana mutations [86, 87], or with the Swedish, Dutch, and London mutations [88]. Differences among the various transgenic mouse models used, the stages of disease progression when neurogenesis was evaluated, and differences in the protocols used to evaluate neurogenesis are factors that might have contributed to the discrepancies reported in the literature [50].

In human AD patients, the expression of several immature neuronal markers (doublecortin (DCX), polysialylated nerve cell adhesion molecule (PSA-NCAM), neurogenic differentiation factor (NeuroD), and *β*III-tubulin) appears to be increased [89], while the expression of the mature neuronal marker microtubule-associated protein (MAP) was found to be dramatically decreased [90] in the DG of the hippocampus. These results suggest that, regardless of an increase in neuronal differentiation, the later stages of neuronal maturation during the neurogenic process might be compromised in the human AD brain. While the exact mechanism responsible for this dysregulation is still unclear, A*β* aggregates have been found to accumulate near neural precursor cells in the hippocampal DG [91, 92] suggesting that these aggregates can influence hippocampal neurogenesis in the AD brain. Furthermore, many of the molecules involved in the development of AD can also play a role during the neurogenic process; for example, PS1 is thought to regulate neuronal differentiation [93], whereas soluble APP*α* may be important during cell proliferation [94].

### 3.2. Hippocampal Neurogenesis in Parkinson's Disease (PD)

PD is caused by death of dopaminergic neurons that project from the substantia nigra (SN) pars compacta to the striatum of the basal ganglia. PD is manifested by (1) severe motor symptoms characterized by a progressive impairment of movement control, akinesia, rigidity, and tremor; and (2) nonmotor symptoms such as cognitive decline, olfactory dysfunction, anxiety [95], and depression [96]. At the neuropathological level, the disease is also characterized by the presence of *α*-synuclein-positive Lewy bodies and dystrophic Lewy neurites throughout the brain, which initially occur in the vagal nerve and OB and thereafter spread to other nuclei and cortical areas [97].

The neurogenic regions of the adult brain are innervated by dopaminergic projections from the SN and the ventral tegmental area (VTA) [98–100]; therefore, the reduction of dopamine (DA) levels that occurs in PD may potentially affect the production of new neurons in the SVZ and DG. Moreover, some of the nonmotor symptoms linked to PD that are not directly associated with neurodegeneration in the SN such as olfactory dysfunction or depression and cognitive alterations [101, 102] may be related to deficits in the stem cell populations of the SVZ/OB system and the hippocampus, respectively [103, 104].

The animal models that have been most widely used in PD research are the unilateral 6-hydroxydopamine (6-OHDA) lesion rat model and the bilateral 1-methyl-4-phenyl-1,2,3,6-tetrahydropyridine (MPTP) lesion mouse model, which develop PD-like symptoms [105]. Park and Enikolopov showed that experimental ablation of dopaminergic neurons in the MPTP mouse model of PD resulted in a transient increase in cell division in the SGZ of the DG [106]. These findings are in agreement with the ones by Peng et al., who reported an increase in the incorporation of BrdU as well as in the number of cells that coexpressed BrdU and the immature neuronal marker DCX in the DG, SVZ, and striatum, but not in the SN of MPTP-treated mice [107]. Despite these results, various studies have also shown a decrease in adult neurogenesis in the SGZ and SVZ of MPTP-treated animals. For example, Höglinger et al. demonstrated that proliferation of C cells (which are targeted by dopaminergic innervations) was impaired both in the SVZ and SGZ of MPTP-treated mice [108]. Furthermore, using both the 6-OHDA and the MPTP models to induce DA depletion in rats and mice, respectively, the same group also found a marked decrease in precursor cell proliferation in both the SGZ of the DG and the SVZ, a deficit that was completely reversed by the administration of the selective agonist of D2-like DA receptors [108, 109], further supporting the idea that the dopaminergic depletion observed in PD brains might result in impaired neurogenesis.

Several* in vivo *studies have also evaluated how hippocampal neurogenesis is altered by the expression of *α*-synuclein. Transgenic mice overexpressing human wild-type *α*-synuclein showed significantly fewer neurons both in the OB as well as in the DG of the hippocampus as compared to their control littermates, an effect that seems to result from a decrease in neuronal precursor survival [110], whereas transgenic mice expressing mutant *α*-synuclein were shown to have impaired hippocampal neurogenesis due to a decrease in proliferation and survival of neural precursor cells [111]. In a different study, Nuber and collaborators also showed reduced hippocampal neurogenesis and cognitive deficits in a conditional *α*-synuclein mouse model. Turning off the transgene expression did halt the progression of these symptoms, although no regression was observed [112].

Finally, a decrease in the number of proliferating cell nuclear antigen (PCNA) positive cells (a marker of cell proliferation) in SVZ and a reduction in the number of nestin- and *β*III-tubulin-positive cells in the DG of the hippocampus have also been found in postmortem tissue from PD patients, presumably as a consequence of dopaminergic denervation of these neurogenic regions [108], providing further evidence of altered hippocampal neurogenesis in the human PD brain.

### 3.3. Hippocampal Neurogenesis in Huntington's Disease (HD)

HD is caused by an expansion of cytosine-adenine-guanine (CAG) trinucleotide repeats in the* HD* gene, which results in an expanded polyglutamine tract in the NH_2_-terminal of the protein huntingtin [113]. In most cases the onset of the disease occurs in midlife, between the ages of 35 and 50 years. The disease progresses over time and is invariably fatal 15 to 20 years after the onset of the first symptoms. Motor disturbances, associated with the loss of voluntary movement coordination, are the classical symptoms of HD, with bradykinesia and rigidity appearing in later stages of the disease. Cognitive capacities are also severely affected during the course of the disease with the slowing of intellectual processes being the first sign of cognitive impairment in HD patients [114]. In fact, deficits in some cognitive functions can in some cases be detected decades before the onset of motor symptoms. These cognitive impairments worsen over time and late-stage HD patients show profound dementia [115–120].

Mutant huntingtin is ubiquitously expressed throughout the organism. However, cell degeneration occurs mainly in the brain, particularly in the striatum and certain layers of the cortex [114, 121]. Nevertheless, cell loss can also be detected in other brain regions, including the hippocampus [121–123], raising the possibility that HD might also be associated with alterations in the endogenous neurogenic capacity.

Several rodent models are currently available to study the effects of the altered* HD* gene. These models primarily differ in the size of the expressed huntingtin fragment, the number of CAG repeats, the promoter driving the transgene, and consequently the expression of the mutant protein, as well as the background strain. As a consequence, each model exhibits unique phenotypes. Nevertheless, most demonstrate progressive neurological phenotypes (e.g., progressive dysfunction in motor ability and cognitive decline) that mimic well the human condition [124–127]. The first studies that analyzed how adult hippocampal neurogenesis is altered in HD used R6/1 [128, 129] and R6/2 [130–132] transgenic HD mice, which express exon 1 of the human* HD* gene (corresponding to approximately 3% of the entire gene) with 115 and 150 CAG repeats, respectively [133], and show cognitive impairments [134, 135]. In both cases a dramatic and progressive reduction in adult hippocampal neurogenesis was found. Of note and in accordance with the faster disease progression characteristic of the R6/2 line [133], a reduction in hippocampal cell proliferation can be detected in these HD mice as early as 2 weeks of age, before the onset of any behavioral abnormalities. This decrease progresses with the course of the disease [131] and by 12 weeks of age (i.e., when animals reach the end stage of the disease), R6/2 mice show a 70% reduction in the number of new cells present in the DG [130]. In agreement with the results obtained with the R6 lines, it has recently been demonstrated that adult hippocampal neurogenesis is also selectively affected in yeast artificial chromosome (YAC) 128 mice [136]. This transgenic mouse model expresses the full-length human* HD* gene with 128 CAG repeats [137] and replicates the slow progression of the human condition [138] while also displaying depressive-like behavior [139] and hippocampal-dependent cognitive deficits [140]. In this study, a significant decrease in cell proliferation, neuronal differentiation, and overall neurogenesis was detected in the DG of early symptomatic to end-stage YAC128 mice [136], once again demonstrating the progressive nature of this neurogenic deficit. Additionally, Kandasamy and colleagues [141] also found a significant and progressive decline in adult hippocampal cell proliferation in a rat model of HD that expresses a truncated cDNA fragment of the HD gene with 51 CAG repeats under the control of the endogenous rat Hdh promotor [142]. Finally, a recent study using knock-in Hdh (Q111) mice, which carry an expanded polyglutamine stretch in the mouse huntingtin protein, has also observed altered DG neuronal maturation along with increased anxiety-like phenotypes [143].

Although it is still unclear how the expression of mutant huntingtin gene might lead to a dysregulation of the neurogenic process, various mechanisms have been proposed to contribute to this disturbance [127]. These include (1) transcriptional dysregulation of key genes known to play a role in neurogenesis such as NeuroD [144]; (2) decreased neurotrophic support, including a reduction in the levels of brain-derived neurotrophic factor (BDNF) [145–152]; as well as (3) deficits in neurotransmission, namely, alterations in the dopaminergic [125, 153] and serotonergic [154–158] systems.

Taken together, these studies support the possible role of mutant huntingtin in disrupting the process of adult hippocampal neurogenesis in the HD brain. These neurogenic deficits can in turn contribute, at least in part, to the cognitive decline and depressive-like symptoms found in HD transgenic models. However, studies in postmortem human HD brains have shown no changes in hippocampal cell proliferation [159] and an actual increase in SVZ neurogenesis [160–163]. Methodological considerations and differences in the numbers of CAG repeats and the levels of expression of the mutant gene might account for the discrepancies observed between the human and the rodent studies [50, 127]. Future studies are thus warranted in order to fully elucidate the role of adult neurogenesis in the development of the cognitive symptoms associated with HD.

## 4. Physical Exercise Prevents Cognitive Decline and Increases Adult Neurogenesis

Even though tremendous advances have been made over the past few decades with regard to our understanding of the etiology of age-related neurodegenerative disorders, to date no effective treatments are available for individuals afflicted with these devastating neurodegenerative diseases. In recent years, physical exercise has emerged as the most effective, low-cost, and low-tech way for successful ageing, and therefore, it has the potential to represent a preventive or disease-slowing therapeutic strategy for age-related neurodegenerative diseases [53].

In support of this hypothesis, a meta-analysis study has shown that 1 to 12 months of exercise in healthy adults brings behavioral benefits, including significant increases in memory, attention, processing speed, and executive function [164]. Moreover, regular engagement in physical exercise in midlife is associated with reduced risks of developing dementia later on in life [52], suggesting that physical exercise might indeed have preventative effects with regard to the development of age-related cognitive decline. In agreement, a prospective observational study has found a reduction in the risk for AD and other forms of dementia in individuals who exercise regularly as compared to those who did not actively engage in physical activity [165].

Evidence from animal studies has suggested that an enhancement in hippocampal neurogenesis may underlie the reported beneficial effects of exercise on cognitive function. Indeed, pioneer studies by van Praag and collaborators showed that physical running not only increased hippocampal neurogenesis [42, 43] but can also improve Morris water maze performance and selectively increase LTP in the DG of three-month-old mice [43]. Thus, in addition to upregulating the neurogenic process, physical activity can also increase the capacity for neurons in the hippocampus to sustain synaptic plasticity and facilitate hippocampal-dependent learning in the same animals. Similarly, in humans three months of physical exercise were shown to correlate with increased blood volume in the DG as assessed by functional magnetic resonance imaging (fMRI) as well as an improvement in cognitive scores [47]. Indeed, exercise is known to increase cerebral blood flow [166], the permeability of the blood brain barrier [167], and angiogenesis [168–171]. Given the possible positive relationship between angiogenesis and neurogenesis found in animal studies [172, 173], the observation that three months of exercise resulted in improved cognition is therefore speculated as a result of increased hippocampal angiogenesis and hence neurogenesis in the human brain [47].

These beneficial effects of physical exercise on cognitive function suggest that exercise might indeed be used as a strategy to prevent cognitive decline in age-related neurodegenerative diseases. Physical exercise has been shown to prevent the age-induced decrease in hippocampal cell proliferation, neurogenesis [174], LTP, and neurotrophin levels [175], as well as enhance hippocampal-dependent learning [55] in aged mice. Moreover, submitting rats to a regime of physical exercise during postnatal development was shown to increase hippocampal neurogenesis and spatial memory later on during adult life [176], highlighting the long-lasting benefits of physical exercise on brain plasticity [176].

The exact unerlying mechanisms of how physical exercise promotes adult hippocampal neurogenesis is still unclear. Neurotrophins such as BDNF, insulin-like growth factor 1 (IGF-1), and vascular endothelial growth factor (VEGF) have been recognized as primary mediators of adult neurogenesis [172, 177–179]. Age-related decline in neurogenesis [21–28] has been associated with decreases in the levels of these trophic factors [180, 181]. Expression of BDNF and IGF-1 genes in hippocampal neurons has been shown in response to exercise training [182]. Both peripheral levels of IGF-1 and VEGF are increased following exercise and enter into the brain by crossing the blood brain barrier [183–185]. VEGF [184] and IGF-1 [183, 186] appear to have an important role in physical exercise-induced hippocampal neurogenesis, since blocking one of these neurotrophic factors substantially diminishes running-induced neurogenesis in rodent studies. Similarly, the knock-out of the BDNF receptor (tyrosine receptor kinase B; TrkB Receptor) in hippocampal progenitor cells diminishes the running-induced increase in hippocampal neurogenesis in mice [187]. Therefore, it is thought that these three neurotrophin factors were suggested to work in concert for mediating exercise-induced hippocampal neurogenesis [188].

## 5. Hippocampal Neurogenesis in Animal Models of Neurodegenerative Diseases following Physical Exercise

Animal models of neurodegenerative diseases constitute valuable tools to unmask the underlying mechanisms by which exercise enhances adult neurogenesis, brain plasticity, and hence cognitive function in the diseased brain (Table 1).

### 5.1. Alzheimer's Disease

Several mouse models of AD have shown that running can promote neurogenesis and cognitive function in the AD brain. Short-term running is able to enhance cognitive function in aged Tg2576 mice [189]. Long-term voluntary running for five months not only decreases extracellular A*β* plaques in the frontal cortex and hippocampus of TgCRND8 AD mice but also enhances their hippocampal-dependent learning in the Morris water maze [190]. Similar results were obtained with the APP/PS1 double-transgenic AD mouse model, where treadmill exercise improved learning and memory function and LTP [191], while also ameliorating some of the neuropathological characteristics of the disease, including a reduction in A*β* deposition and tau phosphorylation as well as a decrease in APP phosphorylation and PS1 expression in the hippocampus [192]. However, since hippocampal neurogenesis was not examined in these studies, it is unclear whether the observed behavioral improvements are linked to an increase in hippocampal neurogenesis in these AD transgenic mice.

On the other hand, studies using the APOE-e4 transgenic mouse model have demonstrated the effect of running on restoring hippocampal plasticity and improving cognitive functions in this AD transgenic mouse model [193–195]. Additionally, the effects of physical exercise on hippocampal neurogenesis have also been evaluated in the APP23 AD transgenic mouse model. In one study, mice were allowed access to a running wheel for 10 days at the ages of 6 and 18 months. In the 6-month-old cohort, proliferation was decreased as compared to control animals and no effect of running was observed. However, at the 18-month time point, a running-induced increase in proliferation and neuronal differentiation was detected in APP23 runners [196], indicating that the AD brain retains the ability to upregulate cell proliferation and neuronal differentiation in response to physical exercise. However, in a different study where APP23 transgenic mice were given access to a running wheel for 11 months starting at 10 weeks of age, the authors failed to detect an increase in cell proliferation and neuronal differentiation in the running group [197]. It is possible that by the time of analysis (i.e., at 17 months of age) the disease progression was already too advanced to allow for detection of any changes in endogenous neurogenesis. Alternatively, these findings might also be a consequence of the well-known age-induced decrease in adult hippocampal neurogenesis [21–26, 28]. However, since previous studies have shown that voluntary physical exercise can still increase hippocampal neurogenesis in wild-type aged mice [55, 174, 198], it is likely that the advancement of the disease was a more prominent factor.

Epidemiological studies have reported a reduced risk of developing dementia in elderlies with higher physical activity [199–201]. Neuroimaging studies indicate that elderlies with higher aerobic fitness have larger hippocampal volumes and perform better on a spatial memory task [202]. Furthermore, a longitudinal study has shown that in cognitively normal adults, participation in greater amounts of physical activity 9 years earlier was associated with greater gray matter volume in several brain areas such as the frontal cortex, parietal cortex, and temporal cortex including the hippocampus, which in turn was associated with a reduced risk of cognitive impairment [203].

Despite the fact that there is abundant evidence suggesting that physical activity might be effective in reducing the risk of developing AD in humans, the exact mechanisms by which physical exercise reduces the risk of AD are still unknown. Animal studies have suggested that physical exercise might result in structural changes in the hippocampus that in turn may reduce the risk for AD future research linking the possible changes of the brain (e.g., changes in hippocampal neurogenesis) with functional outcomes in AD patients or individuals with higher risk for AD will shed light on how physical exercise benefits these individuals.

### 5.2. Parkinson's Disease

Several studies have shown that physical exercise can be beneficial in ameliorating some of the neuropathological and behavioural deficits characteristic of various PD rodent models [204–207]. However, to date only a single study has evaluated how physical exercise modulates the endogenous neurogenic capacity in PD by submitting 6-OHDA-lesioned rats to a regime of treadmill exercise (30 min/day, 5 days/week for 4 weeks) [208]. Forced exercise resulted in the upregulation of the trophic factors BDNF and glial cell-derived neurotrophic factor (GDNF) in the striatum as well as an increase in cell proliferation and the migration of neural stem cells towards the lesion site. Additionally, exercise promoted the preservation of tyrosine hydroxylase (TH; the rate-limiting enzyme during the synthesis of DA) positive fibres in the striatum and TH-positive neurons in the SN [208]. These results suggest that exercise can be a promising noninvasive therapeutic intervention to minimize neuronal degeneration in the PD brain. Despite these promising findings, there are currently no studies evaluating how physical exercise modulates the neurogenic capacity in the DG of the hippocampus of PD rodent models.

A few clinical studies have reported that physical exercise can improve motor function and cognitive performance in human PD patients [209, 210]. There is, however, a paucity of studies addressing the possible interaction among hippocampal neurogenesis, cognitive function, and physical exercise in both lesion and transgenic rodent models of PD. Thus, whether the beneficial effects that physical exercise was shown to have in human PD patients [209, 210] are mediated, at least in part, through a decrease in SN neuronal degeneration and/or an increase in hippocampal neurogenesis is a hypothesis that remains to be elucidated. Nevertheless, these findings suggest that physical exercise may constitute a noninvasive therapeutic option to improve cognition in PD patients.

### 5.3. Huntington's Disease

The use of voluntary physical exercise as a means to promote adult neurogenesis was initially tested in 5-week-old R6/2 HD mice [132]. However, access to a running wheel during an uninterrupted period of 4 weeks was unable to induce an increase in neurogenesis (i.e., cell proliferation and neuronal survival) in these HD transgenic mice. Similarly, running also failed to rescue the deficits in hippocampal neurogenesis observed in R6/1 HD mice [211] and presymptomatic N171-82Q HD mice [212]. Although it is feasible that the cellular pathways underlying the proneurogenic effects of physical exercise might be altered by mutant huntingtin, it is also possible that the development of motor deficits (which appear early on particularly in the R6/2 line [133]) might have incapacitated these mice to actively engage in physical exercise. Additionally, the housing conditions involving social isolation that were employed in some of these studies might have also had a negative impact on the running activity of the mice [211], thus contributing to the ineffective effect of exercise on adult hippocampal neurogenesis.

Nevertheless, other authors have found that exposure of R6/1 mice to physical exercise delayed the onset of rear-paw clasping and improved cognition in adulthood [158], while also delaying the onset of locomotor deficits that can be detected in the juvenile period [213]. In addition, although Pang and collaborators observed that running did not alter the protein levels of the neurotrophin BDNF both in the striatum and the hippocampus of R6/1 HD mice [158], a subsequent study by Zajac and colleagues reported a running-induced increase in* bdnf *gene expression that was specifically observed in R6/1 females but not in their male counterparts [151]. Sex-specific differences in the amount of running the animals engaged in might underlie, at least in part, the dichotic effect that physical exercise had on* bdnf* expression levels in R6/1 females versus males. Of note, it is reasonable to speculate that the inability of physical exercise to consistently upregulate* bdnf *gene expression and protein levels in the hippocampus of R6 mice [151, 158] may be responsible for the lack of proneurogenic effects that was observed in the hippocampus of these HD mice upon exercise [132].

Of note, it has also been reported that R6/1 HD mice show decreases in dendritic spine density and spine length both in striatal and cortical neurons [214]. However, it is unknown whether a similar dendritic pathology could be found in the hippocampus of these HD mice. Nevertheless, it is reasonable to speculate that the running-induced cognitive improvement that was observed in this HD transgenic mouse model [158] may result, at least in part, from structural remodeling of the existing hippocampal neurons. In agreement with this hypothesis, physical exercise is known to increase dendritic complexity, spine density, and synaptic plasticity [43, 215, 216].

In contrast to physical exercise, treatment of R6/1 mice with fluoxetine, a selective serotonin reuptake inhibitor (SSRI) antidepressant, was shown to abolish the impairment in adult hippocampal neurogenesis while also increasing cognitive performance (hippocampal-dependent spatial learning and memory) [155]. These preclinical findings highlight the fact that increasing hippocampal neurogenic capacity in the HD brain might result in improved cognition.

## 6. Assessment of Adult Hippocampal Neurogenesis in Live Human Brain

### 6.1. *In Vivo* Imaging of Neurogenesis

The first evidence for the occurrence of adult neurogenesis in the human brain came from a study by Eriksson and colleagues showing the presence of BrdU-positive cells in postmortem hippocampal and SVZ human tissue obtained from cancer patients that received BrdU injections in life for diagnostic purposes [5]. However, due to technological limitations, it is virtually impossible to evaluate adult neurogenesis in the live human tissue. This has in turn halted the analysis of the functional role of adult neurogenesis in humans. Indeed, the current methods employed to examine adult human neurogenesis mainly rely on immunostaining of postmortem fixed tissues obtained in the clinical setting [5] or on the isolation of human neural progenitor cells from tissue biopsies [17, 18, 217]. However, these methods cannot provide further information on the possible roles of adult neurogenesis during neurodegenerative processes in the human brain.

The development of alternate methods that can be used to assess adult human neurogenesis* in vivo* has emerged as an essential research area within the neurogenesis field. Within this scenario, the recent detection of adult neurogenesis in live human brains using magnetic resonance imaging (MRI) [47] has provided a possible method to discover the functional role of adult neurogenesis in the human brain. In this study, Pereira and colleagues measured cerebral blood volume (CBV, known to correlate with angiogenesis in the brain) as an indirect measure of neurogenesis [47], based on the positive correlation between neurogenesis and angiogenesis reported in animal studies [172, 173]. Additionally, using physical exercise as a well-known upregulator of hippocampal neurogenesis and angiogenesis [55, 218], this group also demonstrated that the increase in CBV was specifically observed in the human hippocampus and correlated with cognitive improvement following a 12-week regime of physical training. The results from human subjects were consistent with the observation that a similar process occurred in mice, where there was a positive correlation between a specific increase in CBV and an increase in the number of BrdU-positive cells in the DG following physical exercise [47]. An alternate* in vivo* imaging method that has been employed to detect neurogenesis in humans consists in using proton nuclear magnetic resonance spectroscopy (^1^H-NMR). This technique uses the magnetic properties of protons to detect a specific biomarker of neural progenitor cells, N-acetylaspartate (NAA, a small metabolite produced by neural progenitor cells), in living tissue [219]. Since these two methods have not yet been validated by other studies so far, further clinical studies would help to validate the feasibility and reliability of using these emerging* in vivo* imaging methods as indirect ways to measure adult neurogenesis in the live human brain.

### 6.2. Peripheral Neurotrophins as Biomarkers for Adult Neurogenesis

Another indirect and noninvasive measure of adult neurogenesis in humans might be the measurement of peripheral biomarkers that correlate well with changes in adult neurogenesis. However, such peripheral biomarkers have not yet been clearly identified.

#### 6.2.1. Brain-Derived Neurotrophin Factor

As mentioned above, BDNF, IGF-1, and VEGF have been recognized as primary mediators of adult neurogenesis [172, 177–179]. Indeed, BDNF is considered to be the most downstream factor mediating the upregulation of hippocampal neurogenesis by exercise [188]. In agreement with this idea, Erickson and colleagues reported that exercise training as a fitness intervention for the aging population effectively attenuates the age-related loss in hippocampal volume while also increasing serum levels of BDNF [220]. Additionally, increases in hippocampal BDNF levels are thought to contribute to the upregulation of adult hippocampal neurogenesis that is observed following antidepressant treatment [221]. Indeed, clinical studies have shown that serum BDNF levels are decreased in depressive patients and that antidepressant treatment can ameliorate this deficit [222].

Given this well-established relationship between various neurotrophins and adult hippocampal neurogenesis, it is reasonable to speculate that the peripheral levels of these trophic factors might be reliable biomarkers of adult hippocampal neurogenesis. However, the exact relationship between peripheral levels of neurotrophins and levels of hippocampal neurogenesis is still unclear. Rachman and colleagues have provided the first evidence that brain BDNF is the major contributor to the increase in plasma BDNF that is observed in response to exercise [223]. Yau and colleagues have also investigated the relationship between levels of hippocampal neurogenesis, plasma neurotrophins levels, and cognitive performance in a rat model of stress. They reported that acute stress-induced enhancement in spatial learning and increase in hippocampal BDNF levels were accompanied by a correspondent increase in plasma BDNF levels. However, this effect was independent of adult hippocampal neurogenesis [224]. Furthermore, exposure to chronic stress significantly decreased hippocampal BDNF levels, neurogenesis, and impaired spatial learning, without affecting plasma BDNF levels [224]. Additionally, a period of 28 days of running was also shown to increase hippocampal neurogenesis and improve spatial learning without significantly changing plasma BDNF levels in rats [224]. Thus, the relationship between peripheral BDNF levels and hippocampal neurogenesis appears to be far from linear, and changes in peripheral levels of BDNF may only be detected upon substantial changes in brain levels of this neurotrophin.

In agreement with the findings from animal studies, a dissociation between central and peripheral BDNF levels has also been shown in the clinical setting. Thus, an increase in the brain levels of BDNF was detected in blood samples from the internal jugular vein following 3 months of endurance training in healthy subjects, but no changes in peripheral BDNF levels were observed in these individuals [225]. Indeed, the responses of plasma or serum BDNF levels to exercise vary considerably among studies, with the majority reporting a transient increase in the plasma/serum levels of this neurotrophin following acute exercise [226]. The timing of blood collection after exercise may contribute to these discrepancies, as elevated BDNF levels seem to return to baseline within 10–60 minutes after exercise and then decrease to a level lower than baseline [226]. In agreement, others have found that peripheral levels of BDNF significantly drop below baseline 2 and 3 hours following acute exercise [227, 228], while a significant decrease in resting serum levels of BDNF was found in trained subjects [229, 230]. Additionally, Lee and colleagues have recently reported a significant reduction in resting serum levels of both BDNF and VEGF in adolescent athletes, who showed improved brain function (specifically in the medial-temporal and frontal areas) when compared to their age-matched controls [231].

#### 6.2.2. Insulin-Like Growth Factor 1

IGF-1 is secreted primarily in the liver [232] and can enter into the brain via transport across the blood-brain and blood-cerebrospinal fluid barriers [233]. Transgenic overexpression of IGF-1 promotes neurogenesis and synaptogenesis in the hippocampus during postnatal development [234]. Furthermore, administration of exogenous IGF-1 (after 6 and 20 days) increases the number of hippocampal proliferative cells [178]. Animal studies have shown that physical exercise could stimulate the release of IGF-1 from the liver and increase the brain uptake and levels of IGF-1 in rodents [186] with a concomitant enhancement of neurogenesis and cognitive function [183].

A positive correlation between serum levels of IGF-1 and cognitive function has also been demonstrated in several clinical studies [235–237]. For example, an increase in peripheral levels of IGF-1 following acute exercise training has been shown in middle-aged men after two trials of 60 min cycling exercise [238] and in road cyclist athletes [239]. However, the exact relationship between changes in IGF-1 levels and hippocampal-dependent cognitive function following acute physical interventions has not yet been elucidated. In contrast to acute exercise, sustained exercise training was shown to have no effect [240] or even a negative effect [241] on IGF-1 levels in healthy subjects. Decreased IGF-1 levels were also found in athletes [231] and subjects after 6 weeks of low intensity cycling [242]. Indeed, the relationship between IGF-1 and sustained physical exercise is equivocal.

#### 6.2.3. Vascular Endothelial Growth Factor

VEGF, a 45 kDa heparin-binding homodimeric glycoprotein, is secreted by skeletal muscle and could be released into the circulation [243]. Acute exercise has been shown to increase levels of VEGF in skeletal muscle [244, 245]. An animal study has demonstrated that expression of VEGF mRNA reaches the peak levels immediately after exercise training and gradually declines within 2 hours and then returns to basal levels within 8 hr [246]. In human muscle, VEGF mRNA expression has been shown to be elevated after 30 min of cessation of exercise [244]. Interestingly, circulating VEGF levels were increased immediately after a marathon run in a moderate-altitude condition [247] but were decreased after a marathon run in high-altitude condition [248]. A different study has also shown that plasma VEGF proteins levels were decreased in the femoral vein following 3 hours of two-legged kicking training, though this training paradigm significantly increased VEGF mRNA levels in the skeletal muscle [249]. Similarly, plasma arterial VEGF is lower following exercise training for 10 days [244]. In contrast, Kraus et al. reported an increase in plasma VEGF levels following acute systemic exercise immediately and 2 hours after exercise in well-trained endurance athletes, but not in sedentary controls with regards to the peak response obtained after exercise. These results suggest that peripheral levels of VEGF are differently affected in trained and sedentary subjects following physical exercise at any time point [250]. However, they found a significant elevation in VEGF levels in both groups.

Voss and colleagues have shown the first link between exercise-induced functional connectivity in the temporal cortex and changes in BDNF, IGF-1, and VEGF in healthy elderlies [251]. They reported that increased temporal lobe connectivity between the bilateral parahippocampus and the bilateral middle temporal gyrus was associated with increased peripheral levels of BDNF, IGF-1, and VEGF in elderlies following 7 weeks of aerobic aerobic walking. Similarly, Lee and colleagues reported a significant improvement of brain function specifically in the frontal and temporal brain regions in teens who regularly exercise when compared to age-matched controls [231]. However, this group observed a negative correlation between neurotrophic factors (BDNF and VEGF) and frontal and medial temporal lobe function. These two studies indicate that the duration of the physical intervention an the age of the individuals may affect how exercise modulate the levels of certain trophic factors.

In conclusion, the relationship between exercise-induced changes in peripheral and central levels of neurotrophic factors has not yet been fully validated, and as such, it is still not feasible to use peripheral levels of neurotrophins as biomarkers for predicting changes in adult neurogenesis in human subjects. Further investigations will be needed to discern the interactions between hippocampal neurogenesis and peripheral and central changes in the levels of neurotrophic factors in animal models and humans, both in basal conditions and following different intervals of physical exercise.

## 7. Conclusion

Several animal studies have provided evidence for a functional role of adult hippocampal neurogenesis in specific forms of hippocampal-dependent learning and memory. The multifactorial nature of adult neurogenesis implies that this complex process can be compromised by a variety of disease conditions and mounting evidence from rodent models over the last two decades suggests that alterations in the normal neurogenic capacity can either contribute to or be a consequence of a wide range of neurological disorders including AD, PD, and HD. Despite some inconsistencies in the literature, there seems to be an overall trend towards a decrease in neurogenesis with neurodegeneration. However, in some cases an upregulation of the endogenous neurogenic function has also been found, which may reflect an intrinsic attempt of the brain to regenerate itself and replace the neurons that are lost during the degenerative process. Furthermore, discrepancies between studies performed in animal models and postmortem human brains are also present in the literature and may reflect differences in the amount of progenitor cell proliferation present in the diseased human brains and the respective rodent models [252].

Nevertheless, the discovery that adult neurogenesis is altered in these chronic neurodegenerative conditions suggests that some of the cognitive deficits associated with these disorders could be caused, at least in part, by these alterations and that therapies aimed at restoring or improving the endogenous neurogenic capacity might be of therapeutic value. As such, various studies have now used rodent models of these disorders to test the potential beneficial effects of therapeutic strategies that are known to promote neurogenesis. In particular, physical activity is a noninvasive and relatively inexpensive strategy that has repeatedly been shown to upregulate adult neurogenesis. Numerous preclinical studies have now demonstrated that these strategies have the potential to mitigate several aspects of the neuropathology and behavioural abnormalities (including cognitive decline) characteristic of various animal models of these disorders while also promoting neurogenesis. Further clinical studies are warranted to further elucidate the exact relationship between adult hippocampal neurogenesis and cognitive decline in various neurodegenerative diseases within the human population. The development and refinement of the current* in vivo* imaging techniques for measurement of adult neurogenesis in the live human brain as well as the discovery of peripheral biomarkers that can be used to determine changes in hippocampal neurogenesis will certainly open new avenues to not only answer these questions but also to diagnose and follow the progression of cognitive decline in various neurodegenerative conditions as well as to measure the effectiveness of treatments aimed at manipulating adult hippocampal neurogenesis. The* in vivo* imaging techniques are promising and applications of these methods in clinical populations with neurodegenerative diseases merit future research to validate their reliability in clinical settings. On the other hand, with emerging knowledge about the functional significance of hippocampal neurogenesis in pattern separation of learning and memory formation, neurogenesis-dependent cognitive tasks (e.g., visual pattern separation task [48]) would be an alternate method for studying alterations in hippocampal neurogenesis in clinical studies.

To conclude, although the exact links between physical exercise, increase adult hippocampal neurogenesis and improved cognition are still unclear due to the current technical limitations, it is undisputable that exercise has a positive impact in the brain both during ageing and neurodegenerative processes that are associated with poor cognitive function including dementia. Therefore, physical exercise has now emerged as the most effective way to delay the aged-related cognitive decline associated with various neurodegenerative diseases. Finally, the development of new pharmacological cognitive enhancers that mimic the effects of physical exercise on the brain may also emerge as a new teherapeutic strategy to prevent cognitive decline in the ageing population.

## Figures and Tables

**Table 1:** Modulation of adult neurogenesis by neurodegenerative diseases and physical exercise.

Neurodegenerative disease	Alteration of adult neurogenesis	Effect of physical exercise on adult neurogenesis
AD		
Rodent models	↑ or ↓ SGZ neurogenesis depending on the transgenic model [78–88]	↑ Learning and memory in various transgenic models [189, 190, 192–195] ↑ or no effect on proliferation and neuronal differentiation in APP23 transgenic mice
Human patients	↑ Proliferation/differentiation in human SGZ from AD patients [89] ↓ Maturation in human SGZ from AD patients [90]	—

PD		
Rodent models	↓ SGZ proliferation in lesion models [108] ↑ SGZ proliferation in MPTP lesion model [107] ↓ SGZ proliferation and survival in *α*-synuclein transgenic mice [111, 112]	Rescue of behavioral deficits in lesion models [175, 204, 205, 207]
Human patients	↓ Proliferation/differentiation in human SGZ from PD patients [108]	↑ Motor and cognitive function in human PD patients [209, 210]

HD		
Rodent models	↓ SGZ neurogenesis in HD transgenic and knock-in models [128–131, 136, 141, 143]	No effect on SGZ neurogenesis in transgenic models [132, 211, 212] ↓ Behavioral and cognitive deficits in transgenic models [158, 213]
Human patients	No changes in cell proliferation in human SGZ from HD patients [159]	—

## Abbreviations

A*β*: Amyloid-*β*
AD: Alzheimer's disease
APO: Apolipoprotein
APP: Amyloid precursor protein
BDNF: Brain-derived neurotrophic factor
BrdU: 5-Bromo–deoxyuridine
CA: Cornus ammonis
CAG: Cytosine-adenine-guanine
CBV: Cerebral blood volume
DA: Dopamine
DCX: Doublecortin
DG: Dentate gyrus
fMRI: Functional magnetic resonance imaging
GABA: Gamma-aminobutyric acid
GDNF: Glial cell-derived neurotrophic factor
GCL: Granule cell layer
^1^H-NMR: Proton nuclear magnetic resonance spectroscopy
HD: Huntington's disease
IGF-1: Insulin growth factor 1
LTP: Long-term potentiation
MAP: Microtubule-associated protein
MPTP: 1-Methyl-4-phenyl-1,2,3,6-tetrahydropyridine
MRI: Magnetic resonance imaging
NAA: N-acetylaspartate
NeuroD: Neurogenic differentiation factor
NFT: Neurofibrillary tangles
NMDA: N-methyl-D-aspartate
OB: Olfactory bulb
PCNA: Proliferating cell nuclear antigen
PD: Parkinson's disease
PS: Presenilin
PSA-NCAM: Polysialylated nerve cell adhesion molecule
RMS: Rostral migratory stream
SGZ: Subgranular zone
SN: Substantia nigra
SSRI: Serotonin reuptake inhibitor
SVZ: Subventricular zone
TH: Tyrosine hydroxylase
TrkB: Tyrosine receptor kinase B
VEGF: Vascular endothelial growth factor
VTA: Ventral tegmental area
YAC: Yeast artificial chromosome
6-OHDA: 6-Hydroxydopamine.

## References

[1] Altman J (1962). Are new neurons formed in the brains of adult mammals?. *Science*.

[2] Altman J, Das GD (1965). Autoradiographic and histological evidence of postnatal hippocampal neurogenesis in rats. *Journal of Comparative Neurology*.

[3] Kaplan MS, Hinds JW (1977). Neurogenesis in the adult rat: electron microscopic analysis of light radioautographs. *Science*.

[4] Cameron HA, Woolley CS, McEwen BS, Gould E (1993). Differentiation of newly born neurons and glia in the dentate gyrus of the adult rat. *Neuroscience*.

[5] Eriksson PS, Perfilieva E, Björk-Eriksson T (1998). Neurogenesis in the adult human hippocampus. *Nature Medicine*.

[6] Gould E, Beylin A, Tanapat P, Reeves A, Shors TJ (1999). Learning enhances adult neurogenesis in the hippocampal formation. *Nature Neuroscience*.

[7] Shors TJ, Miesegaes G, Beylin A, Zhao M, Rydel T, Gould E (2001). Neurogenesis in the adult is involved in the formation of trace memories. *Nature*.

[8] Winocur G, Wojtowicz JM, Sekeres M, Snyder JS, Wang S (2006). Inhibition of neurogenesis interferes with hippocampus-dependent memory function. *Hippocampus*.

[9] Kee N, Teixeira CM, Wang AH, Frankland PW (2007). Preferential incorporation of adult-generated granule cells into spatial memory networks in the dentate gyrus. *Nature Neuroscience*.

[10] Jessberger S, Clark RE, Broadbent NJ (2009). Dentate gyrus-specific knockdown of adult neurogenesis impairs spatial and object recognition memory in adult rats. *Learning and Memory*.

[11] Bruel-Jungerman E, Rampon C, Laroche S (2007). Adult hippocampal neurogenesis, synaptic plasticity and memory: facts and hypotheses. *Reviews in the Neurosciences*.

[12] Lie DC, Song H, Colamarino SA, Ming G-L, Gage FH (2004). Neurogenesis in the adult brain: new strategies for central nervous system diseases. *Annual Review of Pharmacology and Toxicology*.

[13] Kempermann G, Wiskott L, Gage FH (2004). Functional significance of adult neurogenesis. *Current Opinion in Neurobiology*.

[14] Toni N, Teng EM, Bushong EA (2007). Synapse formation on neurons born in the adult hippocampus. *Nature Neuroscience*.

[15] Cameron HA, McKay RD (2001). Adult neurogenesis produces a large pool of new granule cells in the dentate gyrus. *Journal of Comparative Neurology*.

[16] Johansson CB, Svensson M, Wallstedt L, Janson AM, Frisén J (1999). Neural stem cells in the adult human brain. *Experimental Cell Research*.

[17] Kukekov VG, Laywell ED, Suslov O (1999). Multipotent stem/progenitor cells with similar properties arise from neurogenic regions of adult human brain. *Experimental Neurology*.

[18] Roy NS, Wang S, Jiang L (2000). *In vitro* neurogenesis by progenitor cells isolated from the adult human hippocampus. *Nature Medicine*.

[19] Pagano SF, Impagnatiello F, Girelli M (2000). Isolation and characterization of neural stem cells from the adult human olfactory bulb. *Stem Cells*.

[20] Spalding KL, Bergmann O, Alkass K (2013). Dynamics of hippocampal neurogenesis in adult humans. *Cell*.

[21] Ninkovic J, Mori T, Götz M (2007). Distinct modes of neuron addition in adult mouse neurogenesis. *The Journal of Neuroscience*.

[22] Imayoshi I, Sakamoto M, Ohtsuka T, Kageyama R (2009). Continuous neurogenesis in the adult brain. *Development Growth and Differentiation*.

[23] Knoth R, Singec I, Ditter M (2010). Murine features of neurogenesis in the human hippocampus across the lifespan from 0 to 100 years. *PLoS ONE*.

[24] Rao MS, Hattiangady B, Shetty AK (2006). The window and mechanisms of major age-related decline in the production of new neurons within the dentate gyrus of the hippocampus. *Aging Cell*.

[25] Kuhn HG, Dickinson-Anson H, Gage FH (1996). Neurogenesis in the dentate gyrus of the adult rat: age-related decrease of neuronal progenitor proliferation. *The Journal of Neuroscience*.

[26] Kempermann G, Kuhn HG, Gage FH (1998). Experience-induced neurogenesis in the senescent dentate gyrus. *The Journal of Neuroscience*.

[27] Ben Abdallah NM, Slomianka L, Vyssotski AL, Lipp H-P (2010). Early age-related changes in adult hippocampal neurogenesis in C57 mice. *Neurobiology of Aging*.

[28] Gil-Mohapel J, Brocardo PS, Choquette W, Gothard R, Simpson JM, Christie BR (2013). Hippocampal neurogenesis levels predict WATERMAZE search strategies in the aging brain. *PLoS ONE*.

[29] Squire LR (1992). Memory and the hippocampus: a synthesis from findings with rats, monkeys, and humans. *Psychological Review*.

[30] Burgess N (2002). The hippocampus, space, and view points in episodic memory. *Quarterly Journal of Experimental Psychology A*.

[31] Bohbot VD, Corkin S (2007). Posterior parahippocampal place learning in H.M. *Hippocampus*.

[32] James LE, MacKay DG (2001). H.M., word knowledge, and aging: support for a new theory of long-term retrograde amnesia. *Psychological Science*.

[33] Morris RGM (1981). Spatial localization does not require the presence of local cues. *Learning and Motivation*.

[34] Eichenbaum H, Stewart C, Morris RG (1990). Hippocampal representation in place learning. *The Journal of Neuroscience*.

[35] Lassalle JM, Bataille T, Halley H (2000). Reversible inactivation of the hippocampal mossy fiber synapses in mice impairs spatial learning, but neither consolidation nor memory retrieval, in the Morris navigation task. *Neurobiology of Learning and Memory*.

[36] Xavier GF, Costa VC (2009). Dentate gyrus and spatial behaviour. *Progress in Neuro-Psychopharmacology and Biological Psychiatry*.

[37] Snyder JS, Kee N, Wojtowicz JM (2001). Effects of adult neurogenesis on synaptic plasticity in the rat dentate gyrus. *Journal of Neurophysiology*.

[38] Saxe MD, Battaglia F, Wang JW (2006). Ablation of hippocampal neurogenesis impairs contextual fear conditioning and synaptic plasticity in the dentate gyrus. *Proceedings of the National Academy of Sciences of the United States of America*.

[39] Clelland CD, Choi M, Romberg C (2009). A functional role for adult hippocampal neurogenesis in spatial pattern separation. *Science*.

[40] Nakashiba T, Cushman JD, Pelkey KA (2012). Young dentate granule cells mediate pattern separation, whereas old granule cells facilitate pattern completion. *Cell*.

[41] Sahay A, Wilson DA, Hen R (2011). Pattern separation: a common function for new neurons in hippocampus and olfactory bulb. *Neuron*.

[42] van Praag H, Kempermann G, Gage FH (1999). Running increases cell proliferation and neurogenesis in the adult mouse dentate gyrus. *Nature Neuroscience*.

[43] van Praag H, Christie BR, Sejnowski TJ, Gage FH (1999). Running enhances neurogenesis, learning, and long-term potentiation in mice. *Proceedings of the National Academy of Sciences of the United States of America*.

[44] Lemaire V, Koehl M, Le Moal M, Abrous DN (2000). Prenatal stress produces learning deficits associated with an inhibition of neurogenesis in the hippocampus. *Proceedings of the National Academy of Sciences of the United States of America*.

[45] Leuner B, Mendolia-Loffredo S, Kozorovitskiy Y, Samburg D, Gould E, Shors TJ (2004). Learning enhances the survival of new neurons beyond the time when the hippocampus is required for memory. *The Journal of Neuroscience*.

[46] Madsen TM, Kristjansen PE, Bolwig TG, Wörtwein G (2003). Arrested neuronal proliferation and impaired hippocampal function following fractionated brain irradiation in the adult rat. *Neuroscience*.

[47] Pereira AC, Huddleston DE, Brickman AM (2007). An *in vivo* correlate of exercise-induced neurogenesis in the adult dentate gyrus. *Proceedings of the National Academy of Sciences of the United States of America*.

[48] Déry N, Pilgrim M, Gibala M (2013). Adult hippocampal neurogenesis reduces memory interference in humans: opposing effects of aerobic exercise and depression. *Frontiers in Neuroscience*.

[49] Thompson A, Boekhoorn K, van Dam AM, Lucassen PJ (2008). Changes in adult neurogenesis in neurodegenerative diseases: cause or consequence?. *Genes, Brain and Behavior*.

[50] Brocardo P, Patten A, Gil-Mohapel J (2012). Altered adult neurogenesis in neurodegenerative disease. *Neurogenesis Research: New Developments*.

[51] Hillman CH, Erickson KI, Kramer AF (2008). Be smart, exercise your heart: exercise effects on brain and cognition. *Nature Reviews Neuroscience*.

[52] Hamer M, Chida Y (2009). Physical activity and risk of neurodegenerative disease: a systematic review of prospective evidence. *Psychological Medicine*.

[53] Ahlskog JE, Geda YE, Graff-Radford NR, Petersen RC (2011). Physical exercise as a preventive or disease-modifying treatment of dementia and brain aging. *Mayo Clinic Proceedings*.

[54] Yau SY, Lau BW, Tong JB (2011). Hippocampal neurogenesis and dendritic plasticity support running-improved spatial learning and depression-like behaviour in stressed rats. *PLoS ONE*.

[55] van Praag H, Shubert T, Zhao C, Gage FH (2005). Exercise enhances learning and hippocampal neurogenesis in aged mice. *The Journal of Neuroscience*.

[56] Deng W, Aimone JB, Gage FH (2010). New neurons and new memories: how does adult hippocampal neurogenesis affect learning and memory?. *Nature Reviews Neuroscience*.

[57] Santarelli L, Saxe M, Gross C (2003). Requirement of hippocampal neurogenesis for the behavioral effects of antidepressants. *Science*.

[58] Ernst C, Olson AK, Pinel JP, Lam RW, Christie BR (2006). Antidepressant effects of exercise: evidence for an adult-neurogenesis hypothesis?. *Journal of Psychiatry and Neuroscience*.

[59] Yau SY, Lau BW, So KF (2011). Adult hippocampal neurogenesis: a possible way how physical exercise counteracts stress. *Cell Transplantation*.

[60] Lazarini F, Lledo PM (2011). Is adult neurogenesis essential for olfaction?. *Trends in Neurosciences*.

[61] Boccalandro F, Muench A, Salloum J (2004). Interatrial defect sizing by intracardiac and transesophageal echocardiography compared with fluoroscopic measurements in patients undergoing percutaneous transcatheter closure. *Catheterization and Cardiovascular Interventions*.

[62] Barton FE, Ha R, Awada M (2004). Fat extrusion and septal reset in patients with the tear trough triad: a critical appraisal. *Plastic and Reconstructive Surgery*.

[63] Schmidt-Hieber C, Jonas P, Bischofberger J (2004). Enhanced synaptic plasticity in newly generated granule cells of the adult hippocampus. *Nature*.

[64] Bruel-Jungerman E, Davis S, Rampon C, Laroche S (2006). Long-term potentiation enhances neurogenesis in the adult dentate gyrus. *The Journal of Neuroscience*.

[65] Sahay A, Scobie KN, Hill AS (2011). Increasing adult hippocampal neurogenesis is sufficient to improve pattern separation. *Nature*.

[66] Creer DJ, Romberg C, Saksida LM, Van Praag H, Bussey TJ (2010). Running enhances spatial pattern separation in mice. *Proceedings of the National Academy of Sciences of the United States of America*.

[67] Garthe A, Behr J, Kempermann G (2009). Adult-generated hippocampal neurons allow the flexible use of spatially precise learning strategies. *PLoS ONE*.

[68] Tronel S, Belnoue L, Grosjean N (2012). Adult-born neurons are necessary for extended contextual discrimination. *Hippocampus*.

[69] Koehl M, Abrous DN (2011). A new chapter in the field of memory: adult hippocampal neurogenesis. *European Journal of Neuroscience*.

[70] Winner B, Kohl Z, Gage FH (2011). Neurodegenerative disease and adult neurogenesis. *European Journal of Neuroscience*.

[71] Hardy J, Selkoe DJ (2002). The amyloid hypothesis of Alzheimer's disease: progress and problems on the road to therapeutics. *Science*.

[72] Corder EH, Saunders AM, Strittmatter WJ (1993). Gene dose of apolipoprotein E type 4 allele and the risk of Alzheimer's disease in late onset families. *Science*.

[73] Chai CK (2007). The genetics of Alzheimer's disease. *American Journal of Alzheimer's Disease and other Dementias*.

[74] Bentahir M, Nyabi O, Verhamme J (2006). Presenilin clinical mutations can affect *γ*-secretase activity by different mechanisms. *Journal of Neurochemistry*.

[75] Ashe KH (2001). Learning and memory in transgenic mice modeling Alzheimer's disease. *Learning and Memory*.

[76] Mu Y, Gage FH (2011). Adult hippocampal neurogenesis and its role in Alzheimer's disease. *Molecular Neurodegeneration*.

[77] Gil-Mohapel J, Simpson J, Christie BR (2009). Modulation of adult neurogenesis by physical exercise and environmental enrichment: insights for the treatment of neurological disorders. *Adult Neurogenesis and CNS Diseases*.

[78] Haughey NJ, Nath A, Chan SL, Borchard AC, Rao MS, Mattson MP (2002). Disruption of neurogenesis by amyloid *β*-peptide, and perturbed neural progenitor cell homeostasis, in models of Alzheimer's disease. *Journal of Neurochemistry*.

[79] Dong H, Goico B, Martin M, Csernansky CA, Bertchume A, Csernansky JG (2004). Modulation of hippocampal cell proliferation, memory, and amyloid plaque deposition in APPsw (Tg2576) mutant mice by isolation stress. *Neuroscience*.

[80] Verret L, Jankowsky JL, Xu GM, Borchelt DR, Rampon C (2007). Alzheimer's-type amyloidosis in transgenic mice impairs survival of newborn neurons derived from adult hippocampal neurogenesis. *The Journal of Neuroscience*.

[81] Zhang C, McNeil E, Dressler L, Siman R (2007). Long-lasting impairment in hippocampal neurogenesis associated with amyloid deposition in a knock-in mouse model of familial Alzheimer's disease. *Experimental Neurology*.

[82] Donovan MH, Yazdani U, Norris RD, Games D, German DC, Eisch AJ (2006). Decreased adult hippocampal neurogenesis in the PDAPP mouse model of Alzheimer's disease. *Journal of Comparative Neurology*.

[83] Wen PH, Hof PR, Chen X (2004). The presenilin-1 familial Alzheimer disease mutant P117L impairs neurogenesis in the hippocampus of adult mice. *Experimental Neurology*.

[84] Chevallier NL, Soriano S, Kang DE, Masliah E, Hu G, Koo EH (2005). Perturbed neurogenesis in the adult hippocampus associated with presenilin-1 A246E mutation. *The American Journal of Pathology*.

[85] Rodríguez JJ, Jones VC, Tabuchi M (2008). Impaired adult neurogenesis in the dentate gyrus of a triple transgenic mouse model of Alzheimer's disease. *PLoS ONE*.

[86] Jin K, Galvan V, Xie L (2004). Enhanced neurogenesis in Alzheimer's disease transgenic (PDGF-APP_Sw,Ind_) mice. *Proceedings of the National Academy of Sciences of the United States of America*.

[87] López-Toledano MA, Shelanski ML (2007). Increased neurogenesis in young transgenic mice overexpressing human APP(Sw, Ind). *Journal of Alzheimer's Disease*.

[88] Kolecki R, LaFauci G, Rubenstein R, Mazur-Kolecka B, Kaczmarski W, Frackowiak J (2008). The effect of amyloidosis-*β* and ageing on proliferation of neuronal progenitor cells in APP-transgenic mouse hippocampus and in culture. *Acta Neuropathologica*.

[89] Jin K, Peel AL, Mao XO (2004). Increased hippocampal neurogenesis in Alzheimer's disease. *Proceedings of the National Academy of Sciences of the United States of America*.

[90] Li B, Yamamori H, Tatebayashi Y (2008). Failure of neuronal maturation in Alzheimer disease dentate gyrus. *Journal of Neuropathology & Experimental Neurology*.

[91] Buxbaum JD, Thinakaran G, Koliatsos V (1998). Alzheimer amyloid protein precursor in the rat hippocampus: transport and processing through the perforant path. *The Journal of Neuroscience*.

[92] Morys J, Sadowski M, Barcikowska M, Maciejewska B, Narkiewicz O (1994). The second layer neurones of the entorhinal cortex and the perforant path in physiological ageing and Alzheimer's disease. *Acta Neurobiologiae Experimentalis*.

[93] Gadadhar A, Marr R, Lazarov O (2011). Presenilin-1 regulates neural progenitor cell differentiation in the adult brain. *The Journal of Neuroscience*.

[94] Demars MP, Hollands C, Zhao Kda T, Lazarov O (2013). Soluble amyloid precursor protein-alpha rescues age-linked decline in neural progenitor cell proliferation. *Neurobiol Aging*.

[95] Tolosa E, Poewe W (2009). Premotor Parkinson disease. *Neurology*.

[96] Reijnders JS, Ehrt U, Weber WE, Aarsland D, Leentjens AFG (2008). A systematic review of prevalence studies of depression in Parkinson's disease. *Movement Disorders*.

[97] Braak H, Del Tredici K, Rüb U, de Vos RAI, Jansen Steur ENH, Braak E (2003). Staging of brain pathology related to sporadic Parkinson's disease. *Neurobiology of Aging*.

[98] Gasbarri A, Sulli A, Packard MG (1997). The dopaminergic mesencephalic projections to the hippocampal formation in the rat. *Progress in Neuro-Psychopharmacology and Biological Psychiatry*.

[99] Scatton B, Simon H, Le Moal M, Bischoff S (1980). Origin of dopaminergic innervation of the rat hippocampal formation. *Neuroscience Letters*.

[100] Verney C, Baulac M, Berger B, Alvarez C, Vigny A, Helle KB (1985). Morphological evidence for a dopaminergic terminal field in the hippocampal formation of young and adult rat. *Neuroscience*.

[101] Ziemssen T, Reichmann H (2007). Non-motor dysfunction in Parkinson's disease. *Parkinsonism & Related Disorders*.

[102] Haehner A, Boesveldt S, Berendse HW (2009). Prevalence of smell loss in Parkinson’s disease—a multicenter study. *Parkinsonism & Related Disorders*.

[103] Ming GL, Song H (2005). Adult neurogenesis in the mammalian central nervous system. *Annual Review of Neuroscience*.

[104] Zhao Z, Taylor WD, Styner M, Steffens DC, Krishnan KRR, MacFall JR (2008). Hippocampus shape analysis and late-life depression. *PLoS ONE*.

[105] Melrose HL, Lincoln SJ, Tyndall GM, Farrer MJ (2006). Parkinson’s disease: a rethink of rodent models. *Experimental Brain Research*.

[106] Park JH, Enikolopov G (2010). Transient elevation of adult hippocampal neurogenesis after dopamine depletion. *Experimental Neurology*.

[107] Peng J, Xie L, Jin K, Greenberg DA, Andersen JK (2008). Fibroblast growth factor 2 enhances striatal and nigral neurogenesis in the acute 1-methyl-4-phenyl-1,2,3,6-tetrahydropyridine model of Parkinson's disease. *Neuroscience*.

[108] Höglinger GU, Rizk P, Muriel MP (2004). Dopamine depletion impairs precursor cell proliferation in Parkinson disease. *Nature Neuroscience*.

[109] Baker MG, Graham L (2004). The journey: Parkinson's disease. *British Medical Journal*.

[110] Winner B, Lie DC, Rockenstein E (2004). Human wild-type *α*-synuclein impairs neurogenesis. *Journal of Neuropathology & Experimental Neurology*.

[111] Crews L, Mizuno H, Desplats P (2008). *α*-synuclein alters Notch-1 expression and neurogenesis in mouse embryonic stem cells and in the hippocampus of transgenic mice. *The Journal of Neuroscience*.

[112] Nuber S, Petrasch-Parwez E, Winner B (2008). Neurodegeneration and motor dysfunction in a conditional model of Parkinson's disease. *The Journal of Neuroscience*.

[113] MacDonald ME, Ambrose CM, Duyao MP (1993). A novel gene containing a trinucleotide repeat that is expanded and unstableon Huntington's disease chromosomes. *Cell*.

[114] Gil JM, Rego AC (2008). Mechanisms of neurodegeneration in Huntington's disease. *European Journal of Neuroscience*.

[115] Folstein SE, Abbott MH, Chase GA (1983). The association of affective disorder with Huntington's disease in a case series and in families. *Psychological Medicine*.

[116] Folstein SE, Folstein MF (1983). Psychiatric features of Huntington's disease: recent approaches and findings. *Psychiatric Developments*.

[117] Pflanz S, Besson JA, Ebmeier KP, Simpson S (1991). The clinical manifestation of mental disorder in Huntington's disease: a retrospective case record study of disease progression. *Acta Psychiatrica Scandinavica*.

[118] Kirkwood SC, Su JL, Conneally PM, Foroud T (2001). Progression of symptoms in the early and middle stages of Huntington disease. *Archives of Neurology*.

[119] Slaughter JR, Martens MP, Slaughter KA (2001). Depression and Huntington's disease: prevalence, clinical manifestations, etiology, and treatment. *CNS Spectrums*.

[120] Duff K, Paulsen JS, Beglinger LJ, Langbehn DR, Stout JC (2007). Psychiatric symptoms in Huntington's disease before diagnosis: the predict-HD study. *Biological Psychiatry*.

[121] Vonsattel JP, DiFiglia M (1998). Huntington disease. *Journal of Neuropathology & Experimental Neurology*.

[122] Spargo E, Everall IP, Lantos PL (1993). Neuronal loss in the hippocampus in Huntington's disease: a comparison with HIV infection. *Journal of Neurology, Neurosurgery, and Psychiatry*.

[123] Rosas HD, Koroshetz WJ, Chen YI (2003). Evidence for more widespread cerebral pathology in early HD: an MRI-based morphometric analysis. *Neurology*.

[124] Menalled LB, Chesselet MF (2002). Mouse models of Huntington's disease. *Trends in Pharmacological Sciences*.

[125] Hickey MA, Reynolds GP, Morton AJ (2002). The role of dopamine in motor symptoms in the R6/2 transgenic mouse model of Huntington's disease. *Journal of Neurochemistry*.

[126] Levine MS, Cepeda C, Hickey MA, Fleming SM, Chesselet M-F (2004). Genetic mouse models of Huntington's and Parkinson's diseases: illuminating but imperfect. *Trends in Neurosciences*.

[127] Gil-Mohapel J, Simpson JM, Ghilan M, Christie BR (2011). Neurogenesis in Huntington's disease: can studying adult neurogenesis lead to the development of new therapeutic strategies?. *Brain Research*.

[128] Lazic SE, Grote H, Armstrong RJE (2004). Decreased hippocampal cell proliferation in R6/I Huntington's mice. *NeuroReport*.

[129] Lazic SE, Grote HE, Blakemore C (2006). Neurogenesis in the R6/1 transgenic mouse model of Huntington's disease: effects of environmental enrichment. *European Journal of Neuroscience*.

[130] Gil J, Leist M, Popovic N, Brundin P, Petersén Å (2004). Asialoerythropoetin is not effective in the R6/2 line of Huntington's disease mice. *BMC Neuroscience*.

[131] Gil JM, Mohapel P, Araújo IM (2005). Reduced hippocampal neurogenesis in R6/2 transgenic Huntington's disease mice. *Neurobiology of Disease*.

[132] Kohl Z, Kandasamy M, Winner B (2007). Physical activity fails to rescue hippocampal neurogenesis deficits in the R6/2 mouse model of Huntington's disease. *Brain Research*.

[133] Mangiarini L, Sathasivam K, Seller M (1996). Exon I of the HD gene with an expanded CAG repeat is sufficient to cause a progressive neurological phenotype in transgenic mice. *Cell*.

[134] Murphy KP, Carter RJ, Lione LA (2000). Abnormal synaptic plasticity and impaired spatial cognition in mice transgenic for exon 1 of the human Huntington's disease mutation. *The Journal of Neuroscience*.

[135] Nithianantharajah J, Barkus C, Murphy M, Hannan AJ (2008). Gene-environment interactions modulating cognitive function and molecular correlates of synaptic plasticity in Huntington's disease transgenic mice. *Neurobiology of Disease*.

[136] Simpson JM, Gil-Mohapel J, Pouladi MA (2011). Altered adult hippocampal neurogenesis in the YAC128 transgenic mouse model of Huntington disease. *Neurobiology of Disease*.

[137] Slow EJ, van Raamsdonk J, Rogers D (2003). Selective striatal neuronal loss in a YAC128 mouse model of Huntington disease. *Human Molecular Genetics*.

[138] Gil-Mohapel JM (2012). Screening of therapeutic strategies for Huntington's disease in YAC128 transgenic mice. *CNS Neuroscience and Therapeutics*.

[139] Pouladi MA, Graham RK, Karasinska JM (2009). Prevention of depressive behaviour in the YAC128 mouse model of Huntington disease by mutation at residue 586 of huntingtin. *Brain*.

[140] van Raamsdonk JM, Pearson J, Slow EJ, Hossain SM, Leavitt BR, Hayden MR (2005). Cognitive dysfunction precedes neuropathology and motor abnormalities in the YAC128 mouse model of Huntington's disease. *The Journal of Neuroscience*.

[141] Kandasamy M, Couillard-Despres S, Raber KA (2010). Stem cell quiescence in the hippocampal neurogenic niche is associated with elevated transforming growth factor-*β* signaling in an animal model of huntington disease. *Journal of Neuropathology & Experimental Neurology*.

[142] von Hörsten S, Schmitt I, Nguyen HP (2003). Transgenic rat model of Huntington's disease. *Human Molecular Genetics*.

[143] Orvoen S, Pla P, Gardier AM, Saudou F, David DJ (2012). Huntington's disease knock-in male mice show specific anxiety-like behaviour and altered neuronal maturation. *Neuroscience Letters*.

[144] Marcora E, Gowan K, Lee JE (2003). Stimulation of NeuroD activity by huntingtin and huntingtin-associated proteins HAP1 and MLK2. *Proceedings of the National Academy of Sciences of the United States of America*.

[145] Ferrer I, Goutan E, Marín C, Rey MJ, Ribalta T (2000). Brain-derived neurotrophic factor in Huntington disease. *Brain Research*.

[146] Zuccato C, Tartari M, Crotti A (2003). Huntingtin interacts with REST/NRSF to modulate the transcription of NRSE-controlled neuronal genes. *Nature Genetics*.

[147] Canals JM, Pineda JR, Torres-Peraza JF (2004). Brain-derived neurotrophic factor regulates the onset and severity of motor dysfunction associated with enkephalinergic neuronal degeneration in Huntington's disease. *The Journal of Neuroscience*.

[148] Gauthier LR, Charrin BC, Borrell-Pagès M (2004). Huntingtin controls neurotrophic support and survival of neurons by enhancing BDNF vesicular transport along microtubules. *Cell*.

[149] Spires TL, Grote HE, Varshney NK (2004). Environmental enrichment rescues protein deficits in a mouse model of Huntington's disease, indicating a possible disease mechanism. *The Journal of Neuroscience*.

[150] Ciammola A, Sassone J, Cannella M (2007). Low brain-derived neurotrophic factor (BDNF) levels in serum of Huntington's disease patients. *American Journal of Medical Genetics B*.

[151] Zajac MS, Pang TY, Wong N (2010). Wheel running and environmental enrichment differentially modify exon-specific BDNF expression in the hippocampus of wild-type and pre-motor symptomatic male and female Huntington's disease mice. *Hippocampus*.

[152] Ben M'Barek K, Pla P, Orvoen S (2013). Huntingtin mediates anxiety/depression-related behaviors and hippocampal neurogenesis. *The Journal of Neuroscience*.

[153] Petersén Å, Puschban Z, Lotharius J (2002). Evidence for dysfunction of the nigrostriatal pathway in the R6/1 line of transgenic Huntington's disease mice. *Neurobiology of Disease*.

[154] Reynolds GP, Dalton CF, Tillery CL, Mangiarini L, Davies SW, Bates GP (1999). Brain neurotransmitter deficits in mice transgenic for the Huntington's disease mutation. *Journal of Neurochemistry*.

[155] Grote HE, Bull ND, Howard ML (2005). Cognitive disorders and neurogenesis deficits in Huntington's disease mice are rescued by fluoxetine. *European Journal of Neuroscience*.

[156] Duan W, Peng Q, Masuda N (2008). Sertraline slows disease progression and increases neurogenesis in N171-82Q mouse model of Huntington's disease. *Neurobiology of Disease*.

[157] Peng Q, Masuda N, Jiang M (2008). The antidepressant sertraline improves the phenotype, promotes neurogenesis and increases BDNF levels in the R6/2 Huntington's disease mouse model. *Experimental Neurology*.

[158] Pang TY, Stam NC, Nithianantharajah J, Howard ML, Hannan AJ (2006). Differential effects of voluntary physical exercise on behavioral and brain-derived neurotrophic factor expression deficits in huntington's disease transgenic mice. *Neuroscience*.

[159] Low VF, Dragunow M, Tippett LJ, Faull RLM, Curtis MA (2011). No change in progenitor cell proliferation in the hippocampus in Huntington's disease. *Neuroscience*.

[160] Curtis MA, Faull RL, Glass M (2006). A novel population of progenitor cells expressing cannabinoid receptors in the subependymal layer of the adult normal and Huntington's disease human brain. *Journal of Chemical Neuroanatomy*.

[161] Curtis MA, Penney EB, Pearson AG (2003). Increased cell proliferation and neurogenesis in the adult human Huntington's disease brain. *Proceedings of the National Academy of Sciences of the United States of America*.

[162] Curtis MA, Penney EB, Pearson J, Dragunow M, Connor B, Faull RLM (2005). The distribution of progenitor cells in the subependymal layer of the lateral ventricle in the normal and Huntington's disease human brain. *Neuroscience*.

[163] Curtis MA, Waldvogel HJ, Synek B, Faull RLM (2005). A histochemical and immunohistochemical analysis of the subependymal layer in the normal and Huntington's disease brain. *Journal of Chemical Neuroanatomy*.

[164] Smith PJ, Blumenthal JA, Hoffman BM (2010). Aerobic exercise and neurocognitive performance: a meta-analytic review of randomized controlled trials. *Psychosomatic Medicine*.

[165] Blugeot A, Rivat C, Bouvier E (2011). Vulnerability to depression: from brain neuroplasticity to identification of biomarkers. *The Journal of Neuroscience*.

[166] Yancey SL, Overton JM (1993). Cardiovascular responses to voluntary and treadmill exercise in rats. *Journal of Applied Physiology*.

[167] Sharma HS, Cervos-Navarro J, Dey PK (1991). Increased blood-brain barrier permeability following acute short-term swimming exercise in conscious normotensive young rats. *Neuroscience Research*.

[168] Black JE, Isaacs KR, Anderson BJ, Alcantara AA, Greenough WT (1990). Learning causes synaptogenesis, whereas motor activity causes angiogenesis, in cerebellar cortex of adult rats. *Proceedings of the National Academy of Sciences of the United States of America*.

[169] Isaacs KR, Anderson BJ, Alcantara AA, Black JE, Greenough WT (1992). Exercise and the brain: angiogenesis in the adult rat cerebellum after vigorous physical activity and motor skill learning. *Journal of Cerebral Blood Flow and Metabolism*.

[170] Kleim JA, Cooper NR, VandenBerg PM (2002). Exercise induces angiogenesis but does not alter movement representations within rat motor cortex. *Brain Research*.

[171] Swain RA, Harris AB, Wiener EC (2003). Prolonged exercise induces angiogenesis and increases cerebral blood volume in primary motor cortex of the rat. *Neuroscience*.

[172] Jin K, Zhu Y, Sun Y, Mao XO, Xie L, Greenberg DA (2002). Vascular endothelial growth factor (VEGF) stimulates neurogenesis *in vitro* and *in vivo*. *Proceedings of the National Academy of Sciences of the United States of America*.

[173] Louissaint A, Rao S, Leventhal C, Goldman SA (2002). Coordinated interaction of neurogenesis and angiogenesis in the adult songbird brain. *Neuron*.

[174] Kronenberg G, Bick-Sander A, Bunk E, Wolf C, Ehninger D, Kempermann G (2006). Physical exercise prevents age-related decline in precursor cell activity in the mouse dentate gyrus. *Neurobiology of Aging*.

[175] O'Callaghan RM, Griffin ÉW, Kelly ÁM (2009). Long-term treadmill exposure protects against age-related neurodegenerative change in the rat hippocampus. *Hippocampus*.

[176] Gomes da Silva S, Unsain N, Mascó DH (2012). Early exercise promotes positive hippocampal plasticity and improves spatial memory in the adult life of rats. *Hippocampus*.

[177] Zigova T, Pencea V, Wiegand SJ, Luskin MB (1998). Intraventricular administration of BDNF increases the number of newly generated neurons in the adult olfactory bulb. *Molecular and Cellular Neurosciences*.

[178] Åberg MA, Åberg ND, Hedbäcker H, Oscarsson J, Eriksson PS (2000). Peripheral infusion of IGF-I selectively induces neurogenesis in the adult rat hippocampus. *The Journal of Neuroscience*.

[179] Lee J, Duan W, Mattson MP (2002). Evidence that brain-derived neurotrophic factor is required for basal neurogenesis and mediates, in part, the enhancement of neurogenesis by dietary restriction in the hippocampus of adult mice. *Journal of Neurochemistry*.

[180] Lai M, Hibberd CJ, Gluckman PD, Seckl JR (2000). Reduced expression of insulin-like growth factor 1 messenger RNA in the hippocampus of aged rats. *Neuroscience Letters*.

[181] Shetty AK, Hattiangady B, Shetty GA (2005). Stem/progenitor cell proliferation factors FGF-2, IGF-1, and VEGF exhibit early decline during the course of aging in the hippocampus: role of astrocytes. *Glia*.

[182] Ding Q, Vaynman S, Akhavan M, Ying Z, Gomez-Pinilla F (2006). Insulin-like growth factor I interfaces with brain-derived neurotrophic factor-mediated synaptic plasticity to modulate aspects of exercise-induced cognitive function. *Neuroscience*.

[183] Trejo JL, Carro E, Torres-Alemán I (2001). Circulating insulin-like growth factor I mediates exercise-induced increases in the number of new neurons in the adult hippocampus. *The Journal of Neuroscience*.

[184] Fabel K, Fabel K, Tam B (2003). VEGF is necessary for exercise-induced adult hippocampal neurogenesis. *European Journal of Neuroscience*.

[185] Ding YH, Li J, Zhou Y, Rafols JA, Clark JC, Ding Y (2006). Cerebral angiogenesis and expression of angiogenic factors in aging rats after exercise. *Current Neurovascular Research*.

[186] Carro E, Nuñez A, Busiguina S, Torres-Aleman I (2000). Circulating insulin-like growth factor I mediates effects of exercise on the brain. *The Journal of Neuroscience*.

[187] Li Y, Luikart BW, Birnbaum S (2008). TrkB regulates hippocampal neurogenesis and governs sensitivity to antidepressive treatment. *Neuron*.

[188] Cotman CW, Berchtold NC, Christie LA (2007). Exercise builds brain health: key roles of growth factor cascades and inflammation. *Trends in Neurosciences*.

[189] Parachikova A, Nichol KE, Cotman CW (2008). Short-term exercise in aged Tg2576 mice alters neuroinflammation and improves cognition. *Neurobiology of Disease*.

[190] Adlard PA, Perreau VM, Pop V, Cotman CW (2005). Voluntary exercise decreases amyloid load in a transgenic model of Alzheimer's disease. *The Journal of Neuroscience*.

[191] Liu HL, Zhao G, Cai K, Zhao HH, Shi LD (2011). Treadmill exercise prevents decline in spatial learning and memory in APP/PS1 transgenic mice through improvement of hippocampal long-term potentiation. *Behavioural Brain Research*.

[192] Liu HL, Zhao G, Zhang H, Shi LD (2013). Long-term treadmill exercise inhibits the progression of Alzheimer's disease-like neuropathology in the hippocampus of APP/PS1 transgenic mice. *Behavioural Brain Research*.

[193] Nichol K, Deeny SP, Seif J, Camaclang K, Cotman CW (2009). Exercise improves cognition and hippocampal plasticity in *APOEε*4 mice. *Alzheimer's and Dementia*.

[194] Nichol KE, Parachikova AI, Cotman CW (2007). Three weeks of running wheel exposure improves cognitive performance in the aged Tg2576 mouse. *Behavioural Brain Research*.

[195] Nichol KE, Poon WW, Parachikova AI, Cribbs DH, Glabe CG, Cotman CW (2008). Exercise alters the immune profile in Tg2576 Alzheimer mice toward a response coincident with improved cognitive performance and decreased amyloid. *Journal of Neuroinflammation*.

[196] Mirochnic S, Wolf S, Staufenbiel M, Kempermann G (2009). Age effects on the regulation of adult hippocampal neurogenesis by physical activity and environmental enrichment in the APP23 mouse model of Alzheimer disease. *Hippocampus*.

[197] Wolf SA, Kronenberg G, Lehmann K (2006). Cognitive and physical activity differently modulate disease progression in the amyloid precursor protein (APP)-23 model of Alzheimer's disease. *Biological Psychiatry*.

[198] Kannangara TS, Lucero MJ, Gil-Mohapel J (2011). Running reduces stress and enhances cell genesis in aged mice. *Neurobiology of Aging*.

[199] Buchman AS, Boyle PA, Yu L, Shah RC, Wilson RS, Bennett DA (2012). Total daily physical activity and the risk of AD and cognitive decline in older adults. *Neurology*.

[200] Sattler C, Erickson KI, Toro P, Schröder J (2011). Physical fitness as a protective factor for cognitive impairment in a prospective population-based study in Germany. *Journal of Alzheimer's Disease*.

[201] Yaffe K, Barnes D, Nevitt M, Lui L-Y, Covinsky K (2001). A prospective study of physical activity and cognitive decline in elderly women women who walk. *Archives of Internal Medicine*.

[202] Erickson KI, Prakash RS, Voss MW (2009). Aerobic fitness is associated with hippocampal volume in elderly humans. *Hippocampus*.

[203] Erickson KI, Raji CA, Lopez OL (2010). Physical activity predicts gray matter volume in late adulthood: the cardiovascular health study. *Neurology*.

[204] Tillerson JL, Caudle WM, Reverón ME, Miller GW (2003). Exercise induces behavioral recovery and attenuates neurochemical deficits in rodent models of Parkinson's disease. *Neuroscience*.

[205] Zigmond MJ, Cameron JL, Leak RK (2009). Triggering endogenous neuroprotective processes through exercise in models of dopamine deficiency. *Parkinsonism & Related Disorders*.

[206] O'Dell SJ, Gross NB, Fricks AN, Casiano BD, Nguyen TB, Marshall JF (2007). Running wheel exercise enhances recovery from nigrostriatal dopamine injury without inducing neuroprotection. *Neuroscience*.

[207] Fisher BE, Petzinger GM, Nixon K (2004). Exercise-induced behavioral recovery and neuroplasticity in the 1-methyl-4-phenyl-1,2,3,6-tetrahydropyridine-lesioned mouse basal ganglia. *Journal of Neuroscience Research*.

[208] Tajiri N, Yasuhara T, Shingo T (2010). Exercise exerts neuroprotective effects on Parkinson's disease model of rats. *Brain Research*.

[209] Fisher BE, Wu AD, Salem GJ (2008). The effect of exercise training in improving motor performance and corticomotor excitability in people with early Parkinson's disease. *Archives of Physical Medicine and Rehabilitation*.

[210] Petzinger GM, Fisher BE, McEwen S, Beeler JA, Walsh JP, Jakowec MW (2013). Exercise-enhanced neuroplasticity targeting motor and cognitive circuitry in Parkinson's disease. *The Lancet Neurology*.

[211] Ransome MI, Hannan AJ (2013). Impaired basal and running-induced hippocampal neurogenesis coincides with reduced Akt signaling in adult R6/1 HD mice. *Molecular and Cellular Neuroscience*.

[212] Potter MC, Yuan C, Ottenritter C, Mughal M, van Praag H (2010). Exercise is not beneficial and may accelerate symptom onset in a mouse model of Huntington's disease. *PLoS Currents Huntigton Disease*.

[213] van Dellen A, Cordery PM, Spires TL, Blakemore C, Hannan AJ (2008). Wheel running from a juvenile age delays onset of specific motor deficits but does not alter protein aggregate density in a mouse model of Huntington's disease. *BMC Neuroscience*.

[214] Spires TL, Grote HE, Garry S (2004). Dendritic spine pathology and deficits in experience-dependent dendritic plasticity in R6/1 Huntington's disease transgenic mice. *European Journal of Neuroscience*.

[215] Stranahan AM, Khalil D, Gould E (2007). Running induces widespread structural alterations in the hippocampus and entorhinal cortex. *Hippocampus*.

[216] Eadie BD, Redila VA, Christie BR (2005). Voluntary exercise alters the cytoarchitecture of the adult dentate gyrus by increasing cellular proliferation, dendritic complexity, and spine density. *Journal of Comparative Neurology*.

[217] Johansson CB, Momma S, Clarke DL, Risling M, Lendahl U, Frisén J (1999). Identification of a neural stem cell in the adult mammalian central nervous system. *Cell*.

[218] van der Borght K, Kóbor-Nyakas DÉ, Klauke K (2009). Physical exercise leads to rapid adaptations in hippocampal vasculature: temporal dynamics and relationship to cell proliferation and neurogenesis. *Hippocampus*.

[219] Manganas LN, Zhang X, Li Y (2007). Magnetic resonance spectroscopy identifies neural progenitor cells in the live human brain. *Science*.

[220] Erickson KI, Voss MW, Prakash RS (2011). Exercise training increases size of hippocampus and improves memory. *Proceedings of the National Academy of Sciences of the United States of America*.

[221] Duman RS, Nakagawa S, Malberg J (2001). Regulation of adult neurogenesis by antidepressant treatment. *Neuropsychopharmacology*.

[222] Shimizu E, Hashimoto K, Okamura N (2003). Alterations of serum levels of brain-derived neurotrophic factor (BDNF) in depressed patients with or without antidepressants. *Biological Psychiatry*.

[223] Rachman IM, Unnerstall JR, Pfaff DW, Cohen RS (1998). Estrogen alters behavior and forebrain c-fos expression in ovariectomized rats subjected to the forced swim test. *Proceedings of the National Academy of Sciences of the United States of America*.

[224] Yau SY, Lau BW, Zhang ED (2012). Effects of voluntary running on plasma levels of neurotrophins, hippocampal cell proliferation and learning and memory in stressed rats. *Neuroscience*.

[225] Seifert T, Brassard P, Wissenberg M (2010). Endurance training enhances BDNF release from the human brain. *American Journal of Physiology*.

[226] Knaepen K, Goekint M, Heyman EM, Meeusen R (2010). Neuroplasticity exercise-induced response of peripheral brain-derived neurotrophic factor: a systematic review of experimental studies in human subjects. *Sports Medicine*.

[227] Castellano V, White LJ (2008). Serum brain-derived neurotrophic factor response to aerobic exercise in multiple sclerosis. *Journal of the Neurological Sciences*.

[228] Yarrow JF, White LJ, McCoy SC, Borst SE (2010). Training augments resistance exercise induced elevation of circulating brain derived neurotrophic factor (BDNF). *Neuroscience Letters*.

[229] Currie J, Ramsbottom R, Ludlow H, Nevill A, Gilder M (2009). Cardio-respiratory fitness, habitual physical activity and serum brain derived neurotrophic factor (BDNF) in men and women. *Neuroscience Letters*.

[230] Chan KL, Tong KY, Yip SP (2008). Relationship of serum brain-derived neurotrophic factor (BDNF) and health-related lifestyle in healthy human subjects. *Neuroscience Letters*.

[231] Lee TM, Wong ML, Lau BW, Lee JC, Yau SY, So KF (2014). Aerobic exercise interacts with neurotrophic factors to predict cognitive functioning in adolescents. *Psychoneuroendocrinology*.

[232] Butler AA, LeRoith D (2001). Minireview: tissue-specific versus generalized gene targeting of the igf1 and igf1r genes and their roles in insulin-like growth factor physiology. *Endocrinology*.

[233] Pulford BE, Ishii DN (2001). Uptake of circulating insulin-like growth factors (IGFs) into cerebrospinal fluid appears to be independent of the IGF receptors as well as IGF-binding proteins. *Endocrinology*.

[234] O'Kusky JR, Ye P, D'Ercole AJ (2000). Insulin-like growth factor-I promotes neurogenesis and synaptogenesis in the hippocampal dentate gyrus during postnatal development. *The Journal of Neuroscience*.

[235] Arwert LI, Deijen JB, Drent ML (2005). The relation between insulin-like growth factor I levels and cognition in healthy elderly: a meta-analysis. *Growth Hormone and IGF Research*.

[236] Aleman A, de Vries WR, de Haan EH, Verhaar HJJ, Samson MM, Koppeschaar HPF (2000). Age-sensitive cognitive function, growth hormone and insulin-like growth factor 1 plasma levels in healthy older men. *Neuropsychobiology*.

[237] Aleman A, Verhaar HJ, de Haan EH (1999). Insulin-like growth factor-I and cognitive function in healthy older men. *Journal of Clinical Endocrinology and Metabolism*.

[238] Manetta J, Brun JF, Maïmoun L, Fédou C, Préfaut C, Mercier J (2003). The effects of intensive training on insulin-like growth factor I (IGF-I) and IGF binding proteins 1 and 3 in competitive cyclists: relationships with glucose disposal. *Journal of Sports Sciences*.

[239] Zebrowska A, Gasior Z, Langfort J (2009). Serum IGF-I and hormonal responses to incremental exercise in athletes with and without left ventricular hypertrophy. *Journal of Sports Science and Medicine*.

[240] Cearlock DM, Nuzzo NA (2001). Effects of sustained moderate exercise on cholesterol, growth hormone and cortisol blood levels in three age groups of women. *Clinical Laboratory Science*.

[241] Karatay S, Yildirim K, Melikoglu MA, Akcay F, Şenel K (2007). Effects of dynamic exercise on circulating IGF-1 and IGFBP-3 levels in patients with rheumatoid arthritis or ankylosing spondylitis. *Clinical Rheumatology*.

[242] Nishida Y, Matsubara T, Tobina T (2010). Effect of low-intensity aerobic exercise on insulin-like growth factor-I and insulin-like growth factor-binding proteins in healthy men. *International Journal of Endocrinology*.

[243] Gavin TP, Drew JL, Kubik CJ, Pofahl WE, Hickner RC (2007). Acute resistance exercise increases skeletal muscle angiogenic growth factor expression. *Acta Physiologica*.

[244] Gustafsson T, Knutsson A, Puntschart A (2002). Increased expression of vascular endothelial growth factor in human skeletal muscle in response to short-term one-legged exercise training. *Pflügers Archiv*.

[245] Höffner L, Nielsen JJ, Langberg H, Hellsten Y (2003). Exercise but not prostanoids enhance levels of vascular endothelial growth factor and other proliferative agents in human skeletal muscle interstitium. *The Journal of Physiology*.

[246] Breen EC, Johnson EC, Wagner H, Tseng HM, Sung LA, Wagner PD (1996). Angiogenic growth factor mRNA responses in muscle to a single bout of exercise. *Journal of Applied Physiology*.

[247] Schobersberger W, Hobisch-Hagen P, Fries D (2000). Increase in immune activation, vascular endothelial growth factor and erythropoietin after an ultramarathon run at moderate altitude. *Immunobiology*.

[248] Gunga HC, Kirsch K, Röcker L (1999). Vascular endothelial growth factor in exercising humans under different environmental conditions. *European Journal of Applied Physiology and Occupational Physiology*.

[249] Hiscock N, Fischer CP, Pilegaard H, Pedersen BK (2003). Vascular endothelial growth factor mRNA expression and arteriovenous balance in response to prolonged, submaximal exercise in humans. *American Journal of Physiology*.

[250] Kraus RM, Stallings HW, Yeager RC, Gavin TP (2004). Circulating plasma VEGF response to exercise in sedentary and endurance-trained men. *Journal of Applied Physiology*.

[251] Voss MW, Erickson KI, Prakash RS (2013). Neurobiological markers of exercise-related brain plasticity in older adults. *Brain, Behavior, and Immunity*.

[252] Curtis MA, Low VF, Faull RL (2012). Neurogenesis and progenitor cells in the adult human brain: a comparison between hippocampal and subventricular progenitor proliferation. *Developmental Neurobiology*.

